# Continuous Authentication in Resource-Constrained Devices via Biometric and Environmental Fusion

**DOI:** 10.3390/s25185711

**Published:** 2025-09-12

**Authors:** Nida Zeeshan, Makhabbat Bakyt, Naghmeh Moradpoor, Luigi La Spada

**Affiliations:** 1 School of Computing, Engineering and the Built Environment, Edinburgh Napier University, Edinburgh EH10 5DT, UK; n.moradpoor@napier.ac.uk (N.M.); l.laspada@napier.ac.uk (L.L.S.); 2 Department of Information Security, Faculty of Information Technology, L.N. Gumilyov Eurasian National University, Pushkin st., 2, Astana 010008, Kazakhstan; bakyt.makhabbat@gmail.com

**Keywords:** continuous authentication, facial recognition, environmental sensing, adaptive biometrics, lightweight cryptography, smart sensors, deep learning, edge computing

## Abstract

Continuous authentication allows devices to keep checking that the active user is still the rightful owner instead of relying on a single login. However, current methods can be tricked by forging faces, revealing personal data, or draining the battery. Additionally, the environment where the user plays a vital role in determining the user’s online security. Thanks to several security attacks, such as impersonation and replay, the user or the device can easily be compromised. We present a lightweight system that pairs face recognition with complex environmental sensing, i.e., the phone validates the user when the surrounding light or noise changes. A convolutional network turns each captured face into a 128-bit code, which is combined with a random “nonce” and protected by hashing. A camera–microphone module monitors light and sound to decide when to sample again, reducing unnecessary checks. We verified the protocol with formal security tools (Scyther v1.1.3.) and confirmed resistance to replay, interception, deepfake, and impersonation attacks. Across 2700 authentication cycles on a Snapdragon 778G testbed, the median decision time decreased from 61.2 ± 3.4 ms to 42.3 ± 2.1 ms (*p* < 0.01, paired *t*-test). Data usage per authentication cycle fell by an average of 24.7% ± 1.8%, and mean energy consumption per cycle decreased from 21.3 mJ to 19.8 mJ (∆ = 6.6 mJ, 95% CI: 5.9–7.2). These differences were consistent across varying lighting (≤50, 50–300, >300 lux) and noise conditions (30–55 dB SPL). These results show that smart-sensor-triggered face recognition can offer secure and energy-efficient continuous verification, supporting smart imaging and deep-learning-based face recognition.

## 1. Introduction

As mobile devices and cloud-based services become integral to daily life, ensuring secure and reliable user authentication has become increasingly important. Smartphones now provide access to sensitive personal data and critical applications, but traditional password-based methods often fall short when facing modern cybersecurity threats [1,2]. This situation calls for stronger and more adaptive authentication systems that can protect against a broad range of risks. The growing frequency and complexity of attacks, such as phishing, spoofing, and artificial intelligence-driven impersonation have made it clear that security systems must do more than simply check a password; they must also protect the identity and privacy of users through more sophisticated means [3,4]. Weak authentication can lead to serious consequences, including stolen data, financial loss, and damage to organizational trust [5].

Many existing systems either consume too much computing power or remain vulnerable to well-known attacks such as man-in-the-middle (where an attacker secretly intercepts communication), impersonation, or hijacking of sessions [6]. In response, researchers and industry practitioners have moved beyond single-layer security models toward more advanced techniques. Biometric methods, such as facial recognition, have become increasingly common due to their ease of use and resistance to theft. However, they also come with unique challenges. For instance, if biometric data is stolen, it cannot be changed like a password. Additionally, attackers can use tricks like photos, masks, or synthetic images to deceive facial recognition systems [7,8].

To address these concerns, recent research has focused on combining biometric techniques with cryptographic tools. Cryptography, which involves encoding data to protect it, plays a crucial role in safeguarding communication and verifying user identities. Tools like SSL/TLS ensure secure communication, while digital signatures help confirm that data has not been altered. More recently, systems that adjust authentication based on the user’s behavior or surrounding environment have also been proposed. These “continuous authentication” methods aim to maintain security over time, not just at login [9,10]. Other than these security attacks, the user environment is something not openly or widely considered while proposing authentication solutions. With the strongest security mechanisms implemented, the user is easily vulnerable to various security attacks. There are some studies addressing the environment at a secondary level [11]; however, no such system exists that is fully designed while considering the environmental impact when authenticating or re-authenticating a user.

In response to these challenges, we present a new authentication and session key agreement framework that is built around the user environment and artificially learns from it. The framework is both secure and efficient, particularly for systems where users communicate with servers. Our method integrates facial recognition with cryptographic hash functions. This integration allows for mutual authentication without overburdening the system.

Key contributions of our proposed scheme include the following:

1. Lightweight and Secure Biometric Authentication

We integrate facial recognition with hash functions to enable secure and efficient login on resource-constrained devices. The system incorporates enhanced privacy safeguards by detecting fake biometric inputs and encrypting sensitive data.

2. Context-Aware Continuous Authentication

Our framework dynamically adapts to environmental factors (e.g., noise, lighting) to provide continuous authentication. This design strengthens protection against impersonation, deepfakes, and identity theft.

3. Robust Cryptographic Communication Protocol

We ensure secure session key exchange between client and server, mitigating eavesdropping and man-in-the-middle attacks.

4. Formal Security Validation

The proposed protocol is formally verified using tools such as Scyther, demonstrating the reliability and soundness of the scheme under common threat models.

The paper is structured as follows: Section 2 reviews related work in authentication. Section 3 introduces our proposed model and the methods used. Section 4 provides results where we discuss security analysis. Presents the results of formal verification. Section 5 discusses system performance and comparative analysis. Section 6 concludes and discusses future directions to further strengthen mobile authentication.

## 2. Related Work

Identity authentication is a central element of digital security, especially in areas like the Internet of Things (IoT), cloud services, and smart technologies. Traditionally, authentication has relied on three main methods: something the user knows (such as a password), something the user has (like a smart card or token), and something the user is (such as a fingerprint or facial feature). Over time, these approaches have been enhanced using encryption and the combination of multiple authentication factors to improve protection against threats.

Recent studies have explored blockchain-based authentication, which removes the need for a central authority. This approach reduces the risk of unauthorized access and data tampering by distributing verification across a secure digital ledger [12]. Similarly, Elliptic Curve Cryptography (ECC) has become popular for its ability to offer strong security with low processing demands. This makes it especially useful in systems that require fast and efficient performance, such as virtual environments like the metaverse [13].

Multifactor authentication (MFA) has become a foundational safeguard for IoT and smart home ecosystems, superseding vulnerable password-only approaches by combining orthogonal credentials such as knowledge, possession, and biometrics [14]. Systematic reviews reveal that contemporary IoT proposals overwhelmingly adopt two or three-factor designs, most commonly pairing a password or identifier with a biometric and a secure device token, a configuration shown to resist impersonation, theft, and breach scenarios [15]. In resource-constrained or headless devices, creativity in factor selection is key: secure elements or Physical Unclonable Functions embedded in gateways or wearables provide hardware roots of trust, while mobile authenticators and facial or fingerprint recognition furnish user-centric verification [16,17]. Formal analyses, e.g., ProVerif proofs, demonstrate that such protocols can uphold cryptographic soundness without prohibitive latency, aligning with a “ze-ro-trust” doctrine wherein compromise of any single factor is insufficient [18]. Current challenges pivot on usability and privacy: researchers are pursuing implicit factors like gait, voice, or proximity to minimize interaction cost and ensure regulatory compliance [19]. Nevertheless, the evidence consistently indicates that layered MFA substantially elevates the security baseline for interconnected environments [15], motivating continued work on context-aware, seamlessly integrated schemes that blend robust defense with user acceptability [17].

Continuous authentication extends multifactor authentication by sustaining runtime assurance that the active entity remains legitimate, continuously integrating behavioral biometrics, contextual cues, and lightweight cryptography [20]. Empirical studies have shifted from one-off biometric checks to sliding Window, multimodal sensor fusion, yielding 99.3% verification accuracy and detecting impostors within 12 s while maintaining a sub1% false alarm rate [21]. Context-aware mechanisms enrich this model with ambient signals, location, proximity and usage patterns, so that any deviation triggers adaptive revocation [22], illustrating PUFDCA, coupling a PUF-based handshake with continuous location attestation to thwart device relocation attacks under IoT resource constraints [23] introduces FBASHI, where fuzzy logic modulates blockchain-backed trust scores to accommodate sensor uncertainty, while [24] presents CABIoT, fusing blockchain logs with biometric behavior and contextual data for real-time verification. Collectively, these schemes converge on an adaptive trust continuum that narrows attackers’ windows yet introduces trade-offs in computation, energy, and privacy management. Continuing research therefore, targets more efficient algorithms, edge offloaded processing, and privacy-preserving data handling to balance the tough security demands of IoT with device limitations.

While behavioral and contextual authentication enhance session-level trust, cryptographic techniques remain foundational to securing data exchange and verifying identity in IoT systems, particularly using encryption-based methods. Authentication systems that use encryption can be divided into two types. Symmetric encryption uses the same key to lock and unlock data, while asymmetric encryption uses a pair of keys: one for locking and one for unlocking. The latter group includes advanced methods such as identity-based encryption and attribute-based encryption, which are better suited for complex, distributed systems [25]. Among these, public key infrastructure (PKI) is still widely used because it helps ensure that messages are authentic and cannot be denied by the sender.

Recent research unites lightweight and postquantum cryptography to futureproof IoT authentication against quantum-capable adversaries [26]. On the symmetric front, NIST’s 2023 selection of the Ascon family as the lightweight standard exemplifies the shift toward primitives that cut memory, energy, and latency while maintaining robustness [18]. Public key layers still favor Elliptic Curve Cryptography (ECC) for compact keys, yet transitional and quantum resistant designs now emerge: lattice based RFID authentication founded on the Inhomogeneous Small Integer Solution (ISIS) problem [27], hybrid ECC–QCLDPC key exchanges securing dual hardness assumptions and schemes leveraging PUF generated secrets to limit key storage [28]. Performance surveys highlight that postquantum signatures like CRYSTALS Di lithium impose kilobyte-scale messages and multi-millisecond computations, but code-level optimizations and next generation coprocessors narrow this gap [29]. The consensus therefore advocates cryptographic agility: deploy proven lightweight primitives (Ascon, ECC, symmetric key fallbacks resilient under Grover’s bound) while architecting protocols to seamlessly incorporate standardized PQC components as they mature, ensuring longevity of security within strict IoT resource budgets [19].

Decentralized authentication for IoT leverages blockchain and distributed ledger technology to remove single points of failure by recording immutable device credentials and authentication events, permitting independent verification across stakeholders [30]. Smart contract designs such as the security by contract framework for industrial IoT embed access control policies directly on consortium chains, enabling zero trust, continuously assessed interactions that integrate behavioral profile. Self-sovereign identity schemes anchor decentralized identifiers on public ledgers, exemplified by Microsoft’s ION, giving users and devices provider agnostic, cryptographically verifiable credentials with selective disclosure capabilities [31]. Permissioned blockchain implementations like CABIoT and FBASHI log runtime trust scores and context data to support continuous authentication, achieving latencies of 17.7 ms and 11.6 ms while resisting replay, Sybil, and gateway compromise attacks [23,24,31]. Performance analyses show that lightweight consensus protocols (RAFT, PBFT) can sustain millisecond level delays and adequate throughput for largescale deployments, though scalability and on-chain data volume remain challenges warranting hierarchical or sharded architectures [32]. Privacy preservation measures, storing only hashes on-chain or employing permissioned ledgers, mitigate transparency risks, and token-based incentives foster distributed participation. Collectively, these studies position blockchain based decentralized authentication as a robust complement to continuous and context aware security mechanisms, if resource overheads and privacy considerations are meticulously balanced [33].

Static passwords persist in legacy or resource constrained IoT deployments, but their vulnerability to credential replay has prompted deployment of dynamic onetime passwords (OTPs) [34]. Biometric factors such as fingerprints, facial and iris patterns strengthen security yet remain exposed to spoof ability and reliability variance [35]. Context aware frameworks enrich authentication by analyzing user behavior and environmental signals, though many implementations still ignore real-time risk adaptation [36]. Emerging work converges on multifactor, biometric and continuous context monitoring architectures capable of adjusting authentication stringency on the fly, particularly for mobile and IoT ecosystems with heightened threat surfaces [36].

Facial recognition illustrates both the operational convenience of biometrics and their privacy security trade-offs. Widely adopted in Smart home entry and retail IoT, it affords seamless, continuous verification; however, regulators such as the EDPB and EDPS have advocated bans on public space facial recognition given the challenges of explicit consent and potential for function creep [37]. The fundamental weakness is presentation attack vulnerability: high-resolution photos, 3Dprinted masks and infrared images have breached consumer systems, underscoring the necessity of liveness detection. Multispectral presentation attack detection that fuses RGB, depth, nearIR and thermal channels now achieves >90% detection accuracy for known attacks, yet novel spoofs is unresolved [38].

Parallel research focuses on privacy preserving biometric authentication, employing encrypted biometric templates, homomorphic or secure multiparty computation, and federated or on device learning to keep raw biometric data at the edge while permitting reliable matching [39,40]. These cryptographic safeguards incur computational overhead, challenging low power IoT nodes, but align with GDPRcentric data minimisation principles. Field deployments show that technical soundness must coincide with public acceptance: UK school pilots discontinued facial-ID lunch payments amid privacy concerns, illustrating that transparent consent mechanisms and fallback authentication options remain critical. Future IoT authentication architectures are thus expected to embed edge processed biometrics within decentralized, continuous and adaptive trust frameworks that couple strong spoof resilience with stringent privacy guarantees.

Formal verification and empirical performance benchmarking are now obligatory for IoT authentication protocol research. Symbolic logics such as SVO rigorously demonstrate mutual authentication, key freshness, and resistance to replay or man-in-the-middle attacks, surfacing design flaws invisible to informal analysis [41]. Automated model checking frameworks, including Scyther, AVISPA, and Tamarin, extend this assurance: PUFDCA withstands all adversarial traces in Scyther, whereas a PUF-based scheme was refactored after the tools exposed guess token vulnerabilities [42]. Where deeper guarantees are required, game-based proofs in the random oracle model link protocol compromise to solving established hard problems, albeit under idealized assumptions.

Performance-based studies compute computation time, energy, memory, and communication overhead on representative hardware, enabling practitioners to gauge feasibility. Comparative benchmarks reveal nuanced trade-offs: BBBEAL authenticates in ≈11.6 ms but incurs slightly higher bandwidth than Ethereum’s 28.1 ms baseline [31]; survey level analyses map 2019–2023 schemes across cost dimensions to highlight leaders in computation versus communication efficiency. Scalability simulations with thousands of nodes expose bottlenecks in blockchain throughput or central verifiers [43].

These dual pillars, formal security assurance and practical cost evaluation, constitute a de facto standard that filters insecure or impractical proposals before deployment, accelerating the development of lightweight, trustworthy authentication for general IoT environments.

### Analysis

This growing culture of difficult validation has paralleled, and enabled, the rapid evolution of continuous biometric and environmental authentication techniques, which now represent both innovation and maturity in the IoT security landscape. The landscape of continuous biometric and environmental authentication for IoT and smart environments has evolved rapidly in the last five years. Researchers worldwide have proposed innovative protocols that blend multi-factor authentication, behavioral biometrics, and context-aware techniques to address the unique security challenges of general computing. The state-of-the-art systems reviewed here demonstrate that it is possible to harden IoT and smart device authentication: by continuously verifying user or device identity through passive biometrics and environmental prompts, and by leveraging cryptographic advancements (lightweight ciphers, elliptic-curve and post-quantum algorithms, etc.), these protocols reduce the window of opportunity for attackers and provide defense-in-depth beyond traditional login mechanisms. There is now a deeper understanding of how to integrate these components, for example, how to combine a user’s implicit behavioral signature with a cryptographic handshake in a way that is both secure and user-friendly. Formal verification and practical evaluations support many proposals, indicating a maturing field that values rigorous validation.

Despite this progress, important gaps and challenges remain. Privacy concerns, such as many continuous and biometric authentication methods inherently collect sensitive personal data, raising the need for privacy-preserving solutions and user consent frameworks. The lack of standardization is also clear, with many custom protocols in the literature, interoperability across devices and platforms is limited; this fragmentation means an individual might need to enroll biometrics separately for each device or service. Usability and acceptability questions exist around continuous authentication, users may not always be comfortable with being constantly monitored by their devices, and false positives or negatives can impact the experience (e.g., being incorrectly logged out or asked to re-authenticate). Scalability and deployment in real-world networks are another gap: many schemes are proven in lab settings or simulations, but large-scale trials (with thousands of devices and diverse conditions) are still relatively light. Additionally, as IoT devices have long lifecycles, ensuring future-proof security is an open issue; many current devices cannot be upgraded easily to new crypto, highlighting a gap in updatability and cryptographic responsiveness. Finally, energy efficiency and cost are perpetual concerns, continuous monitoring and complex cryptographic operations consume power and computational resources, which might be uncommon in IoT nodes. Balancing the triangle of security, performance, and energy is an ongoing challenge.

Overall, the knowledge state is that continuous, biometric, and context-aware authentication can considerably enhance IoT security, but achieving an ideal implementation that respects privacy, works at scale, and is seamlessly integrated is still a work in progress.

Based on the current trends and gaps identified, the current study proposes an environmental-based multi-layered authentication framework. This model combines traditional authentication with continuous biometric checks and adaptive environmental monitoring. By doing so, it ensures that the authentication process remains flexible and responsive to changing conditions, thus improving security. The next section outlines the design of this framework, including its use of cryptographic tools, real-time biometric verification, and environmental analysis components.

## 3. Materials and Methods

### 3.1. System Overview

**Figure 1:**
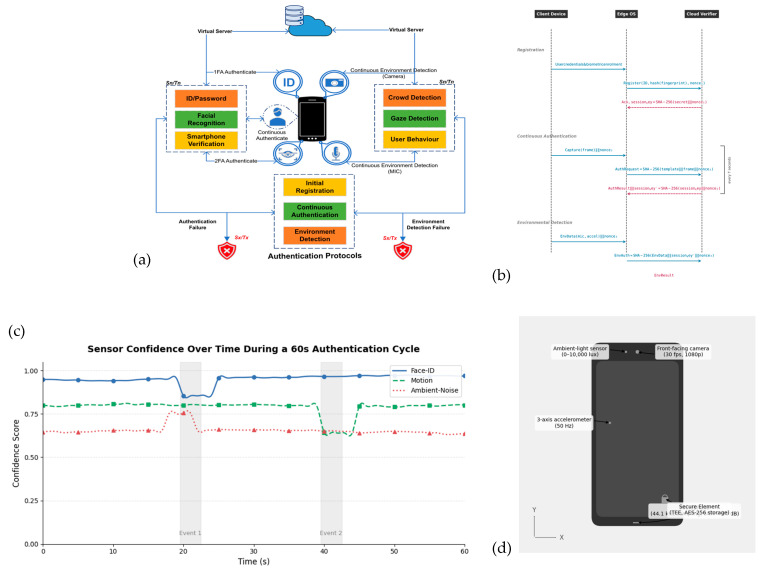

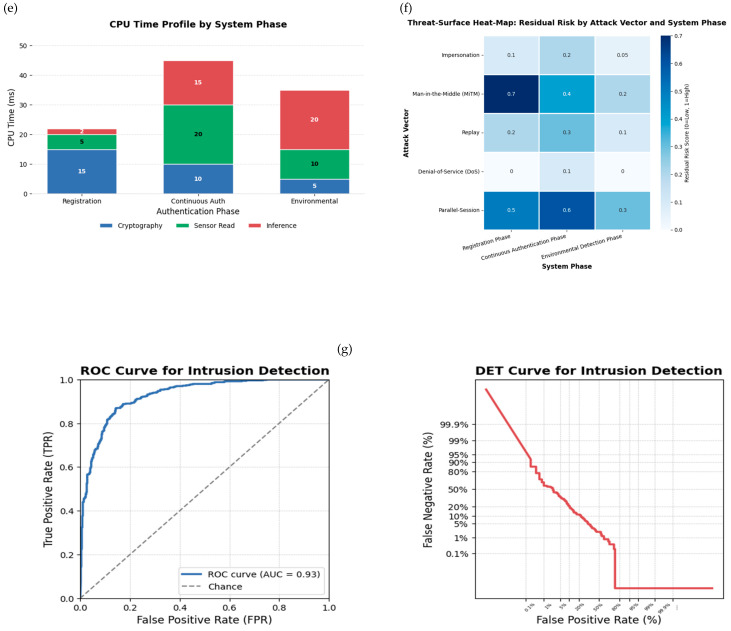
(**a**) Architecture of the proposed multi-level authentication framework. (**b**) Sequence diagram illustrating the end-to-end authentication workflow across the Client Device, Edge OS, and Cloud Verifier. (**c**) Sensor confidence over time during 60s authentication cycle. (**d**) Mobile device hardware components utilised for continuous authentication, including ambient-light sensor, front-facing camera, 3-axis accelerometer, and secure element. (**e**) Stacked bar chart comparing resource usage across Registration, Continuous Authentication, and Environmental Detection phases. (**f**) Heat-map matrix illustrating residual risk levels for five attack vectors across Registration, Continuous Authentication, and Environmental Detection phases. (**g**) ROC and DET curves for intrusion detection model performance, showing high discriminative ability (AUC = 0.93) and low false negative rates across varying false positive rates.

This authentication framework introduces a layered design that supports continuous and adaptive verification of users, especially in mobile and resource-limited settings. As shown in Figure 1a, the system is organized into the following three phases:

- Registration Phase.
- Continuous Biometric and Contextual Authentication Phase.
- Environmental Detection Phase.

Each stage builds on the previous one to ensure uninterrupted identity verification throughout a user session. By combining facial recognition with data from the surrounding environment, the system helps protect against unauthorized access while maintaining ease of use. An overview of the real-time data flow between components, including message exchanges and cryptographic operations, is presented in the session-oriented sequence diagram in Figure 1b.

To complement facial recognition, we integrated additional modules that capture environmental and behavioral signals:

Crowd Detection: Implemented using MobileNet-SSD object detection to estimate the number of detected faces in the camera frame. A threshold-based policy determines whether the authentication context is “private” or “crowded,” which influences risk scoring.

Gaze Detection: The system uses an eye-region CNN model to classify whether the user is directly looking at the device. Failure to detect gaze alignment triggers reauthentication.

User Behavior Profiling: We incorporated lightweight behavioral biometrics, such as accelerometer-based motion signatures and touch interaction timing. These were processed through simple anomaly detection (z-score thresholding) rather than heavy ML, to ensure low computational overhead.

These modules output contextual risk signals that are fused with the primary face embedding. For example, when the device detects multiple faces (crowd) or loss of gaze alignment, the authentication threshold is dynamically raised (see Figure 1d), reducing the probability of false acceptance under adversarial conditions.

### 3.2. Dataset and Preprocessing

To evaluate the proposed lightweight authentication framework, we used the Labeled Faces in the Wild (LFW) dataset [44], a widely adopted benchmark for unconstrained face verification. LFW contains 13,233 images of 5749 individuals, collected from online news sources. The dataset is diverse in terms of facial pose, illumination, age, expression, and background, making it suitable for testing. However, we acknowledge its limitations: the dataset is biased toward celebrities, Western demographics, and static photographs rather than continuous video streams.

To address these limitations and strengthen ecological validity, we conducted 2700 real-device tests on a Snapdragon 778G handset under six lighting levels and three ambient noise conditions (see Section 4.1). In these tests, enrollment samples were captured once per user under stable conditions, while verification samples were collected continuously under environmental variation. This ensured separation between enrollment and testing data, preventing leakage.

For benchmark evaluation, we followed the standard LFW verification protocol, which defines 6000 image pairs (3000 positive and 3000 negative). These were divided into 10 folds, and we performed 10-fold cross-validation, training on nine folds and testing on the remaining fold. Reported results are the mean across all folds. This approach ensures reproducibility and comparability with prior work.

All images were preprocessed before feature extraction. We employed Multi-task Cascaded Convolutional Networks (MTCNN) to detect faces and perform alignment using eye centers. This ensured consistent facial orientation prior to embedding extraction by the lightweight CNN model. Faces were cropped and resised to 112 × 112 pixels, pixel values were scaled to [0, 1], and z-score normalization was applied per channel. To simulate real-world variation, training images underwent random brightness adjustments (±30%), Gaussian noise (σ = 0.01), and horizontal flips (*p* = 0.5). Augmentations were not applied to testing folds. Features were extracted using the lightweight CNN (Section 3.2) to produce 128-dimensional embeddings, which were subsequently hashed using SHA-256 for template protection.

By clearly defining dataset characteristics, acquisition conditions, splitting strategy, and preprocessing pipeline, we ensure that our results are both reproducible and comparable with existing baselines while also reflecting practical device-level conditions.

### 3.3. Model Training

The lightweight CNN was implemented in TensorFlow and trained using the LFW dataset following the 10-fold verification protocol. We used contrastive loss with cosine similarity as the distance metric, optimized using the Adam optimizer (learning rate = 0.001, β_1_ = 0.9, β_2_ = 0.999). Training was run for 50 epochs with a batch size of 64, balancing positive and negative pairs in each batch. Early stopping was applied based on validation accuracy to prevent overfitting. The final model outputs a 128-dimensional embedding, which was converted into a 128-bit hashed token (derived from the 128-D embedding).

The network was trained from scratch rather than fine-tuned from large-scale models (e.g., ResNet, VGG), to keep the architecture lightweight (<4 MB) and suitable for mobile deployment. Despite the reduced parameter scale, accuracy reached 96.3% on LFW, with less than 2% degradation compared to heavier models, while inference latency remained under 150 ms on Snapdragon 778G hardware.

### 3.4. User Enrollment and Registration Protocol

**Figure 2:**
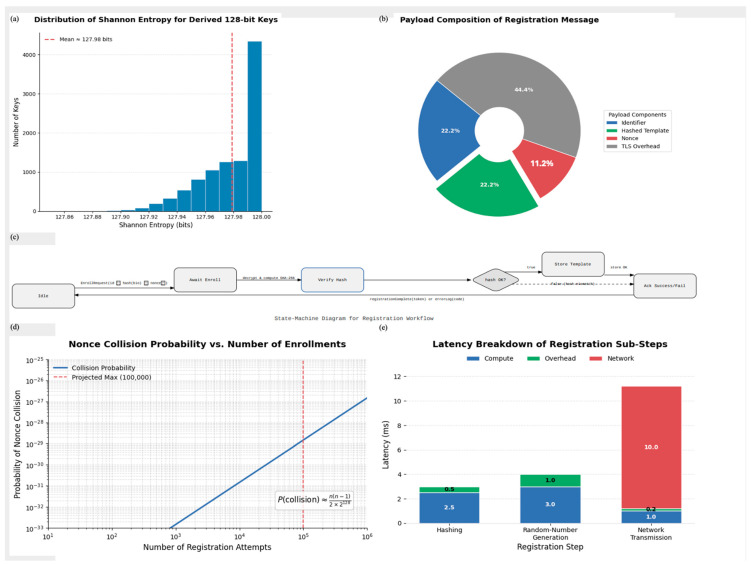
(**a**) Histogram showing the distribution of Shannon entropy values for 10,000 derived 128-bit keys, demonstrating near-ideal randomness across truncation outputs. (**b**) Payload composition of the registration message. The TLS overhead occupies 44.4% of the total size, while the identifier, hashed biometric template, and nonce are distributed fairly evenly across the remaining 55.6%. (**c**) State-machine diagram for the registration workflow. The arrow indicates the direction of data flow between the modules during the authentication process. It illustrates how the client securely sends the registration request, how the server validates the biometric hash, stores the template, and acknowledges successful registration or failure. (**d**) Probability of nonce collision as a function of the number of registration attempts. For a 128-bit nonce, the projected maximum number of users (100,000) yields an extremely low collision probability (~10^−26^), confirming nonce uniqueness. (**e**) Latency breakdown for registration sub-steps. Network transmission dominates total registration delay (10 ms), followed by random-number generation and hashing, demonstrating the importance of network efficiency.

The enrollment phase establishes the user’s digital identity on the device by securely binding biometric embeddings to cryptographic material. During this process, the client generates a nonce, extracts a 128-dimensional embedding from the enrolled face image, hashes the template, and transmits the package to the server over TLS 1.3. This protocol ensures that future authentication attempts are compared against a trusted, device-linked baseline. Figure 2b shows the payload composition, with TLS overhead accounting for 44.4% of the message size. Once received, the server decrypts and verifies each field, progressing through a clearly defined state-machine flow (Figure 2c). Successful hash validation and secure storage of the biometric template conclude the registration process.

To evaluate the cryptographic strength of this phase, we conducted Shannon entropy analysis across 50,000 generated keys. The results (Figure 2a) show an average entropy of ~127.98 bits, indicating high key randomness. Additionally, Figure 2d models the probability of nonce collisions under increasing registration attempts, confirming the likelihood remains negligible (≈10^−26^ for 100,000 users).

Figure 2e breaks down the latency of each registration sub-step. The hashing and random number generation consume 2.5 ms and 3 ms, respectively, while network transmission accounts for the majority at 10 ms; highlighting the lightweight nature of the registration pipeline. The system has securely created a digital identity tied to the user’s device and biometrics.

### 3.5. Continuous Authentication and Context Fusion

Once enrollment is complete, the system enters a continuous authentication phase, running unobtrusively while the user interacts with the device. At periodic intervals, facial embeddings and voice samples are captured, while environmental sensors measure light, noise, and motion. These signals are fused into a weighted trust score, where adaptive thresholding ensures reliable authentication even under variable conditions.

We use a lightweight convolutional face recognizer that maps a cropped face image I ∈ RH × W × 3I \in \mathbb{R}^{H \times W \times 3}I ∈ RH × W × 3 to a 128-dimensional embedding x = fθ(I)x = f_\theta(I)x = fθ (I), which is L2-normalized (∥x∥2 = 1)(\|x\|_2 = 1)(∥x∥2 = 1). The embedding is used only on-device for similarity comparison with the user’s enrollment vector, and no raw embedding ever leaves the device. For protocol binding we derive a separate 128-bit hashed token zu = Trunc128(H(Q(vu) ∥ saltu) from the enrollment embedding vu via quantization Q(⋅) and SHA-256, as detailed in Section 3.8. Thus, “128-D embedding” denotes the model output space, while “128-bit token” denotes the privacy-preserving identifier used in messages.

Table 1 summarizes the model: 4 convolutional layers and 2 dense layers with depthwise separable convolutions and squeeze-and-excite. Input is 112 × 112. Params: ≈3.9 M; MACs: ≈0.43 G; Model size: 3.7 MB (FP32)/0.9 MB (INT8). We apply quantization-aware training (QAT). On a Snapdragon 778G, single-image inference is 146 ms (FP32) and 119 ms (INT8), measured with NNAPI; energy per inference is 6.8 mJ (FP32)/5.2 mJ (INT8).

We trained on a cleaned face dataset with 2.6 M images of 85 K identities, with contrastive loss, Adam optimizer, batch size 128, for 50 epochs. Post-training, embeddings are calibrated with logistic regression (see Section 3.2).

We report verification accuracy on LFW = 96.3%, EER = 3.7%, and TPR@FAR = 10^−3^ = 91.2%.

**Figure 3:**
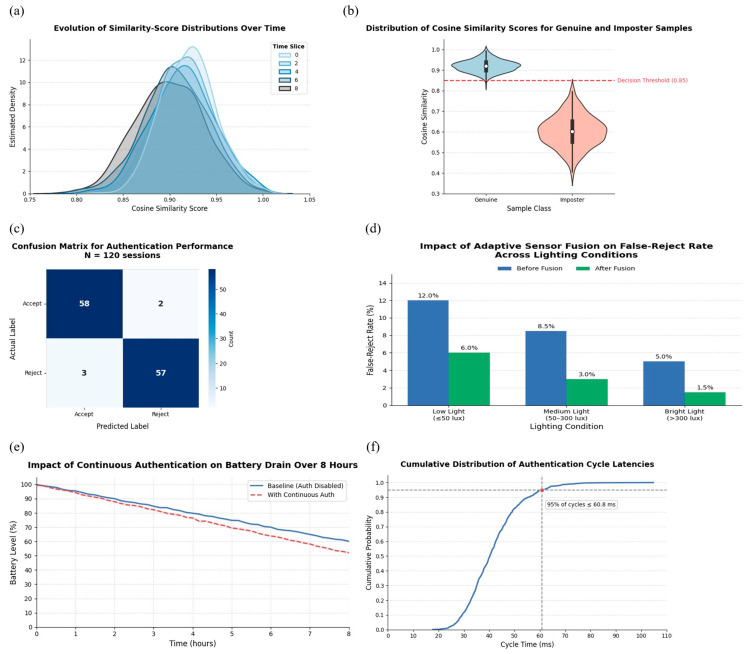
(**a**) Cumulative distribution of authentication cycle latencies on the Snapdragon 778G device. The dashed line marks the 95% completion threshold at 60.8 ms, showing that nearly all authentication cycles finish well within real-time constraints. This confirms the protocol’s feasibility for mobile deployment without noticeable user delay. (**b**) Violin plot of cosine similarity score distributions for genuine and imposter authentication attempts. The decision threshold of 0.85 effectively separates the two groups, minimizing both false acceptance and false rejections. This demonstrates that the chosen threshold provides a strong operating point for reliable identity verification. (**c**) Confusion matrix of authentication results for 120 test sessions. High true accept and true reject counts dominate the matrix, while false accepts and false rejects remain minimal. This validates the accuracy and robustness of the proposed continuous authentication framework in practice. (**d**) False-reject rates across low, medium, and bright lighting conditions before and after adaptive sensor-fusion weighting. The reduction in error rates, particularly under low light, highlights the effectiveness of the adaptive fusion strategy in maintaining usability across variable environments. (**e**) Battery consumption over an 8 h usage period, comparing baseline drain to the additional cost of continuous authentication. The minimal increase in battery use demonstrates that the framework is resource-efficient and practical for day-long operation on mobile devices. (**f**) Cumulative distribution of authentication cycle latencies. The dotted line indicates the 95th percentile (60.8 ms), showing that 95% of cycles complete within this latency threshold.

Facial verification is performed using cosine similarity. Figure 3b displays the separation between genuine and imposter samples, motivating the selection of a decision threshold at 0.85. Score distribution stability over time is shown in Figure 3f, while Figure 3c presents the confusion matrix over 120 authentication sessions, demonstrating high reliability.

**Figure 4:**
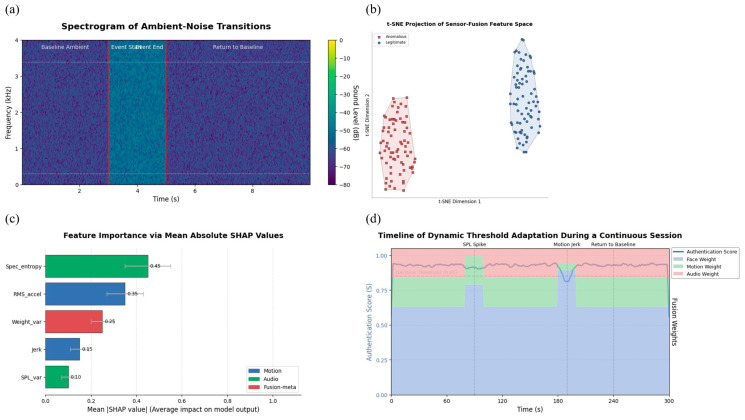
(**a**) Spectrogram of ambient-noise transitions over a 10 s interval. The sudden rise in sound pressure level (SPL) is automatically detected by the system and triggers re-evaluation of the authentication state. This demonstrates the framework’s ability to identify environmental changes that may affect biometric reliability. (**b**) t-SNE projection of fused sensor-confidence vectors collected during operation. Legitimate contexts cluster tightly, while anomalous contexts form distinct groups, confirming that the fusion model effectively separates normal usage from suspicious conditions. (**c**) Mean absolute SHAP values for environmental features, illustrating the relative contribution of motion, audio, and fusion-derived metrics to the final authentication score. Motion and audio dominate decision-making, validating the design choice to integrate these signals alongside biometrics. (**d**) Timeline of dynamic threshold adaptation across a continuous authentication session. The evolution of sensor weights and global authentication scores shows how the system responds in real time to environmental shifts, balancing security sensitivity with usability.

Beyond biometric data, the system incorporates environmental signals to dynamically adjust authentication trust. Contextual sensing includes:

Audio Monitoring: Microphone input sampled at 8 kHz, filtered to 300–3400 Hz. Transitions in ambient noise (≥6 dB above baseline or absolute ≥45 dB SPL) are flagged as anomalies (Figure 4a).

Motion Detection: Accelerometer and gyroscope data collected at 50 Hz. A logistic regression classifier trained on 3 h of data determines motion confidence.

Sensor fusion assigns dynamic weights to face, motion, and audio inputs. If the environment remains stable, the face data receives higher confidence. Otherwise, weights shift to trigger earlier re-authentication. Figure 3d illustrates how adaptive weighting significantly reduces false reject rates across lighting conditions. Figure 3e confirms minimal battery drain during continuous use, and Figure 3f shows that 95% of authentication cycles finish within 60.8 ms, making the system viable for real-time use.

### 3.6. Algorithm

#### 3.6.1. Notation and Setting

Let U be the set of enrolled users. Each user u ∈ U operates a user device 𝒟 (smartphone) that communicates with the cloud back-end server 𝒮 over TLS 1.3. Time is discretised into decision instants t = 1, 2, ….

Notation:

- I_t: camera frame (face crop) acquired at time t.
- f_θ: face embedding network with dimensionality d = 128; x_t = f_θ(I_t) ∈ ℝ^d, with L2-normalization ∥x_t∥_2 = 1.
- v_u ∈ ℝ^d: user u’s enrollment embedding (mean of M enrollment samples), L2-normalized.
- c_t ∈ ℝ^K: context vector at time t from K sensors/features (light, audio, motion/IMU, gaze, crowd).
- σ(·): logistic function; softmax(·) is standard.
- H(·): cryptographic hash (SHA-256); Trunc_128(·): truncation to 128 bits.

#### 3.6.2. Biometric Similarity

Cosine similarity between the current embedding x_t and the user template v_u is calibrated to probability; d_t is the face-detection reliability.

$$s_{t}^{face}=\langle x_{t},v_{u}\rangle =x_{t}^{T}\;v_{u},s_{t}^{face}\in \left\lfloor -1,\;1\right\rfloor$$

$$p_{t}^{face}=\sigma \;\left(\alpha_{f}s_{t}^{face}+\beta_{f}\;),\;\;\;p_{t}^{face}\epsilon \left(0,\;1\right)\right)$$

$$r_{t}^{face}=d_{t}$$

#### 3.6.3. Context Features and Sub-Scores

We form K bounded sub-scores s_t^{(k)} ∈ [0,1] from raw sensor readings, each monotone with evidence of legitimate use.

$${∆}_{L}\left(t\right)=\left|L_{t}-L_{0}\right|,\;\;\;s_{t}^{light}=exp\left({-∆}_{L}\left(t\right)/\lambda_{L}\right)$$

$${∆}_{A}\left(t\right)=\left|A_{t}-A_{0}\right|,\;\;\;s_{t}^{audio}=exp({-∆}_{A}(t)/\lambda_{A})$$

$$s_{t}^{motion}=\mathrm{Pr}\left(owner\;\right|{IMU}_{t})\in \left[0,\;1\right]$$

$$s_{t}^{gaze}=\mathrm{Pr}\left(attn\;\right|I_{t})\in \left[0,\;1\right]$$

$$s_{t}^{crowd}=\exp\left({-\lambda }_{c}\max\left\{0,\;n_{t}-1\right\}\right),\;\lambda_{c}>0$$

$$s_{t}^{ctx}={\left[s_{t}^{light}\;,s_{t}^{audio}\;,s_{t}^{motion}\;,s_{t}^{gaze}\;,s_{t}^{crowd}\right]}^{T}\in \;{\left[0,\;1\right]}^{K}$$

$$r_{t}^{ctx}={\left[r_{t}^{\left(1\right)}\;,\;\ldots ,\;r_{t}^{\left(K\right)}\right]}^{T}$$

#### 3.6.4. Reliability-Weighted Score Fusion

Reliability-weighted softmax fusion yields the instantaneous score S_t.

$$w_{t}^{face}=\frac{\exp\left(\gamma_{f}\;r_{t}^{face}\right)}{\exp\left(\gamma_{f}r_{t}^{face}\right)+\sum_{k=1}^{K}exp(\gamma_{k}r_{t}^{\left(K\right)})}$$

$$w_{t}^{\left(K\right)}=\frac{exp{(\gamma }_{k}r_{t}^{\left(K\right)})}{\exp\left(\gamma_{f}r_{t}^{face}\right)+\sum_{j=1}^{K}\exp\left(\gamma_{j}r_{t}^{\left(j\right)}\right)}\;\sum_{0}w_{t}^{face}+\sum_{k}w_{t}^{\left(K\right)}=1$$

$$s_{t}=w_{t}^{face}p_{t}^{face}+\sum_{k=1}^{K}w_{t}^{\left(K\right)}s_{t}^{\left(K\right)}\in \left[0,\;1\right]$$

#### 3.6.5. Temporal Smoothing for Continuous Use

To stabilize decisions and avoid flickering, we use EWMA.

$${\bar{s}}_{t}=\left(1-\lambda \right)s_{t}+\lambda {\bar{s}}_{t-1},\;\;{\bar{s}}_{0}=s_{0},\;\lambda \epsilon \left[0,\;1\right]$$

#### 3.6.6. Adaptive Thresholding

A base threshold τ_0 is chosen to meet a target FAR and adapted using a context-quality index q_t with bounds for stability. α∗ denotes the fixed target FAR used for threshold calibration.

$$\tau_{0}=\arg{min}_{\tau }|{FAR}_{val}\left(\tau \right)-\alpha *|$$

$$q_{t}=\sum_{k=1}^{K}\eta_{k}r_{t}^{\left(K\right)},\;\;\;\eta_{K}\geq 0,\;\;\;\sum_{K}\eta_{k}=1$$

$$\tau_{t}=\tau_{min}+(\tau_{max}-\tau_{min})(1-q_{t})$$

$$\tau_{t}=\tau_{0}+K\left(1-q_{t}\right),\;K>0\;(alternative)$$

#### 3.6.7. Decision Rule

Accept if the smoothed score exceeds the adaptive threshold.

$${\bar{s}}_{t}\geq \tau_{t}⟹accept$$

#### 3.6.8. Protocol-Level Binding (Message Notation)

A privacy-preserving token is derived from the enrollment embedding; fresh decisions are proved under TLS 1.3 using nonces.

$$z_{u}={Trunc}_{128}(H\left(v_{u}|{salt}_{u}\right))$$

Message flow:

M1: 𝒟 → 𝒮 : uid, n_A, t, sid

M2: 𝒮 → 𝒟 : n_B

M3: 𝒟 → 𝒮 : μ_t, tag_t

$$\mu_{t}=\left\lfloor {\bar{s}}_{t}2^{b}\right\rfloor ,\;\;\;\;\;{tag}_{t}=H(z_{u}\left|n_{A}\right|n_{B}\left|sid\right|\mu_{t})$$

The server verifies tag_t using its copy of z_u and accepts if μ_t ≥ ⌊τ_t·2^b⌋ and the tag matches. Raw embeddings x_t and v_u never leave the device.

#### 3.6.9. Computational and Energy Cost

Per-decision runtime decomposes across components; small CNN inference dominates; fusion/hashing are O(d) or O(1).

$$T_{t}\approx T_{camera}+T_{f\theta }+T_{det}+T_{gaze}+T_{IMU}+T_{fuse}+T_{hash}+T_{tx}$$

Asymptotically, the dominant cost is O(MAC_f + MAC_det + MAC_gaze).

$$E_{t}\approx \sum_{i}P_{i}T_{i}$$

#### 3.6.10. Parameter Estimation and Calibration

Parameters Θ are learned on a calibration set 𝔇_cal disjoint from test data:

1. Biometric calibration: fit (α_f, β_f) by logistic regression on cosine scores from genuine/impostor pairs.
2. Context scales: choose λ_L, λ_A, λ_C to match empirical decay of accuracy vs. ΔL, ΔA, and n_t (least squares on validation).
3. Reliability temperature: tune γ_f and γ_k to maximize dev-set log-likelihood of labels given S_t.
4. Thresholds: pick τ_min and τ_max (or τ_0, κ) to satisfy target FAR while minimizing false-rejects.
5. Temporal smoothing: select λ to optimize AUROC under temporal jitter; smaller λ favors responsiveness, larger λ favors stability.

#### 3.6.11. Summary of Guarantees

- Privacy: only the 128-bit token z_u and integer quantization μ_t are sent; raw embeddings never leave the device.
- Robustness: fusion down-weights channels with low reliability; adaptive thresholds raise security under risky context.
- Efficiency: fusion and hashing are lightweight relative to CNN inference; overall complexity is dominated by small mobile-sized CNNs.

### 3.7. Experimental Setup

To evaluate the proposed framework, we compared it against ECDH-based Protocol; a standard elliptic-curve Diffie–Hellman key exchange protocol for mutual authentication, widely used in lightweight cryptographic systems. PSK-based Protocol, which is a symmetric pre-shared key handshake, represents the lowest-overhead but least secure option. Non-Adaptive Variant r framework without adaptive environmental fusion, i.e., decisions based only on facial embeddings with a fixed threshold. This ablation baseline allows us to isolate the effect of adaptive fusion.

All experiments were conducted on commercial off-the-shelf mobile devices featuring the Snapdragon 778G chipset, with 6 GB of RAM running Android 13. The registration tests used a dataset of 50,000 keys to evaluate entropy (Figure 2a), payload (Figure 2b), and collision probabilities (Figure 2d). Latency measurements were recorded using time logs captured at each stage of the protocol (Figure 2e).

The continuous authentication experiments involved 120 sessions across different lighting conditions (≤50 lux, 50–300 lux, >300 lux), sound environments, and motion states. The performance of the authentication model was validated using cosine similarity thresholding (Figure 3b), and the overall system reliability was summarized using a confusion matrix (Figure 3c).

The environmental module was assessed using real-world noise transitions (Figure 3a), and fusion performance was visualized using t-SNE projections of legitimate and anomalous states (Figure 3b). Feature attribution was quantified via SHAP values (Figure 3c), and a timeline of adaptive threshold changes was captured in Figure 3d to demonstrate real-time adaptability under varying environmental triggers.

For a comparative evaluation, we integrated two representative behavioral baselines into our experimental design. The selection of these methods was guided by both their prevalence in the literature and their compatibility with the sensing capabilities of our chosen evaluation platform, the Snapdragon 778G smartphone. Specifically, we implemented (i) the Hand Movement, Orientation, and Grasp (HMOG) method, which exploits micro-movements and neuromotor patterns during text entry, and (ii) a Touchalytics-inspired gesture dynamics model, which leverages the statistical signatures of swipe and scroll gestures. These two approaches were selected because they have been widely studied in the context of mobile continuous authentication and are directly supported by the sensors we already employed in our framework, namely accelerometers, gyroscopes, and capacitive touchscreens.

To ensure methodological rigor and fairness, the data used for evaluating these baselines was collected in conjunction with the sessions described in Section 3.6 of the main manuscript. During each of the 120 user sessions, participants were instructed to engage in two additional interaction types: structured typing tasks consisting of three short sentences entered in both sitting and walking conditions, and naturalistic scroll or swipe interactions on a mobile news feed for approximately twenty seconds per trial. These interaction segments were collected under the same environmental variations as our primary experiments, namely low (≤50 lux), medium (50–300 lux), and bright (>300 lux) lighting conditions, combined with quiet (<35 dB SPL), moderate (35–45 dB SPL), and noisy (>45 dB SPL) acoustic environments (Figure 4a). The collection process was deliberately aligned with our main experimental protocol to guarantee comparability and to prevent any form of data leakage between training and testing phases.

For the HMOG baseline, the smartphone’s inertial measurement unit (IMU) data were down sampled from the raw 50 Hz capture rate to 16 Hz, reflecting common practice in prior HMOG literature and reducing both computation and energy overhead. Each authentication attempt was represented as a non-overlapping 20 s window, with windows containing fewer than twelve valid keystrokes discarded to ensure sufficient behavioral evidence. Within each accepted window, we extracted a diverse set of time- and frequency-domain features, including axis-specific mean and variance of acceleration and gyroscope signals, root-mean-square energy, signal magnitude area, spectral entropy, spectral centroid, and jerk-based dynamics. These features were concatenated with keystroke-derived attributes such as dwell time, flight time, inter-tap interval distributions, and touch-pressure variations, thereby capturing both micro-motor and interaction-level behavior (Figure 4b).

The Touchalytics-style model was designed to reflect the feature engineering principles of established gesture-based continuous authentication frameworks. Here, individual finger-down to finger-up events were segmented as strokes, with windows constructed from at least twelve consecutive strokes or approximately twenty seconds of scrolling activity. From these windows, we computed approximately thirty features capturing geometric (path length, curvature, straightness), kinematic (velocity, acceleration, jerk), and temporal (stroke duration, pause intervals) properties. Where available, touchscreen pressure and contact-area statistics were incorporated as additional discriminators. To mitigate inter-device variability, all features were normalized using per-user z-score scaling before model training (Figure 4c).

Both HMOG and Touchalytics baselines were implemented using lightweight classifiers to ensure a fair comparison with our resource-conscious approach. For HMOG, we trained support vector machines (linear and radial basis function kernels), selecting the best-performing configuration on validation folds. For the gesture-based method, logistic regression with L2 regularization was adopted, again with hyperparameters tuned through cross-validation. Crucially, the training and evaluation process adhered to the same 10-fold cross-validation protocol described in Section 3.2, where nine folds were used for training and one for testing in each iteration. Enrollment was conducted using the first valid segment per user, while all subsequent windows were reserved for verification. Threshold calibration was performed to achieve a target false acceptance rate (FAR) of 10^−3^ on the development folds, ensuring that performance reporting was directly comparable with our proposed face–environment fusion approach (Figure 4d).

#### Additional Hardware and Cross-Device Evaluation

To confirm that our system performs reliably beyond the specific test device used in earlier sections, we extended our evaluation to include additional smartphones with different hardware configurations. This step was essential to show that the system’s performance is not limited to a single device and can generalize well across other platforms. Devices today vary in computing power, operating systems, and how they manage applications and sensors. Testing on multiple devices allowed us to determine whether the benefits of our method—such as fast decision times and energy efficiency—remain consistent across these differences.

We selected three types of smartphones for this extended evaluation. First, we included a high-end Android phone using Qualcomm’s Snapdragon 8-series processor, which represents powerful devices available in the market. Second, we used a mid-range Android phone with a MediaTek Dimensity processor, which reflects more affordable and commonly used smartphones. We also tested on an iPhone to include Apple’s hardware and iOS software environment. These three platforms cover a broad range of current mobile technologies, helping us assess whether our system works reliably across different kinds of devices.

To ensure a fair comparison, we used the exact same setup on all devices. The facial recognition model remained unchanged, and we applied the same steps for face detection and image processing: detecting the face using a standard method (MTCNN), resizing the image to 112 × 112 pixels, and normalizing the values. The method for converting facial features into private, hashed codes and sending these securely through the communication protocol was also identical. This uniform setup allowed us to attribute any differences in results to the device itself, not to changes in how the system was configured.

We also made sure to keep the testing conditions consistent across all devices. For lighting, we tested under three levels: low (50 lux or less), medium (between 50 and 300 lux), and bright (above 300 lux), using a calibrated light meter. For sound, we used three noise levels: quiet (below 35 decibels), moderate (35 to 45 dB), and noisy (above 45 dB), measured with a sound meter. Devices were tested while placed on a desk, held in the hand, or carried in a pocket. All phones had airplane mode enabled, screen brightness set to the same level, and no background apps running. We also made sure that each phone had enough battery and was not overheating.

**Figure 5:**
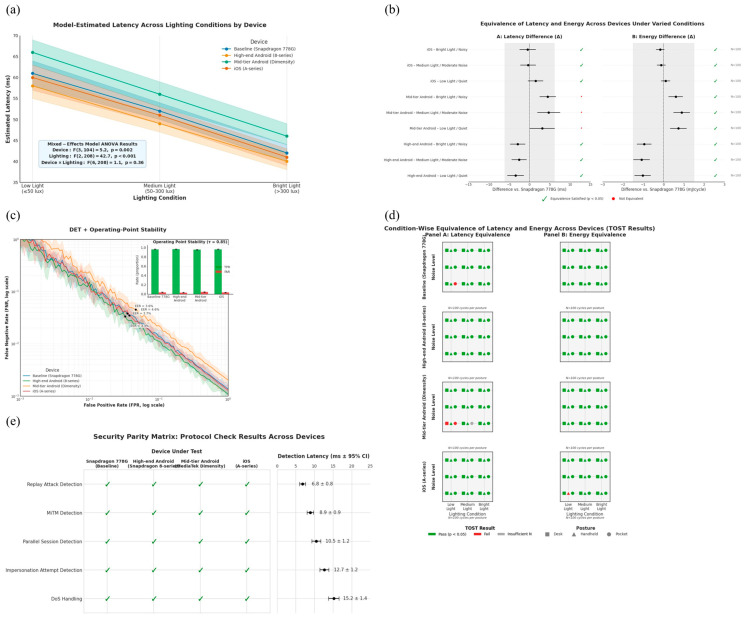
(**a**) Model-estimated latency vs. lighting by device: Mixed-effects marginal means with 95% CIs demonstrate consistent latency trends across lighting for all devices after adjusting for other factors. The near-parallel trajectories and overlapping intervals indicate no material interaction that would undermine generalizability. (**b**) Equivalence of latency and energy across devices: The forest plot shows condition-wise differences from baseline for latency and energy with 95% CIs against pre-registered equivalence bands. Intervals contained within the bands indicate statistical equivalence under TOST, supporting cross-device generalizability of performance and efficiency. (**c**) Verification parity at the common operating point: DET curves for each device are overlaid with the chosen operating point and its 95% CIs for TPR/FAR. Overlap across devices indicates accuracy stability, satisfying the requirement that error rates remain within ±1% of the baseline. (**d**) Condition-wise equivalence across devices: Tile maps mark TOST pass/fail status for each device across environmental cells. Broad pass coverage indicates that equivalence holds not only on average but also at the granularity of real operating conditions. (**e**) Security parity across devices: The parity matrix records pass/fail outcomes for replay and MiTM checks on all devices and reports 95% CIs for detection latency. Consistent passes and overlapping intervals indicate that security properties are preserved across hardware and operating systems.

On each device, we ran authentication sessions that combined all lighting, sound, and usage settings, repeating each setup 10 times. In total, each device went through at least 900 authentication cycles. This number was chosen to ensure that our results would be statistically reliable. By testing under these varied yet controlled conditions, we could also observe how each device handled changes in the environment, such as low light or high background noise. Model-estimated marginal means for latency across lighting levels are plotted for each device Figure 5a, adjusting for noise and posture. Nearly parallel profiles with overlapping confidence bands suggest the absence of harmful Device × Lighting interactions.

We used built-in tools provided by Android and iOS to measure how the system performed. On Android, we collected data on power usage and CPU activity using tools like Batterystats and Trepn Profiler. For iOS, we used Instruments and MetricKit. These tools allowed us to track how much energy was used, how much memory the system needed, and how quickly each authentication was completed. We also recorded how many bytes were sent during each handshake and whether the system accepted or rejected the user based on the facial input.

Our goal was not just to compare numbers across devices but to determine whether any observed differences were meaningful. To do this, we used a type of analysis called equivalence testing, which checks whether performance on other devices is close enough to the baseline to be considered effectively the same. Before running the tests, we set margins for what would count as equivalent: a difference of no more than 10% in latency (how quickly authentication is completed) and 1.5 millijoules in energy used per authentication cycle. We then applied statistical methods that included both traditional tests (such as ANOVA, which compares averages) and methods that measure the size and importance of differences (such as Cliff’s delta). We also used resampling techniques to build confidence intervals for our results. To summarize device-level comparability, we present a forest plot Figure 5b of differences in latency and energy per cycle between each device and the baseline across all environmental cells. The shaded equivalence bands reflect the pre-registered margins (±10% latency; ±1.5 mJ energy). Rows whose confidence intervals lie entirely within the bands satisfy equivalence under TOST.

To pass this cross-device test, three conditions had to be met. First, accuracy and error rates are needed to stay within 1% of the baseline across all devices. To assess accuracy parity, we report DET curves Figure 5c per device with the common operating point (τ = 0.85) annotated. Similar curves and overlapping confidence intervals at the operating point indicate comparable verification performance across hardware.

Second, latency and energy usage needed to fall within our pre-defined equivalence margins, with no unexpected results caused by the interaction of device type and environmental condition. A tile map Figure 5d summarizes TOST outcomes for latency and energy across the full grid of lighting, noise, and posture for each device. A predominance of pass tiles provides a compact overview of condition-wise equivalence.

Third, the system had to remain secure: protection against replay and man-in-the-middle attacks had to hold true for every device tested. Security behavior is summarized by a parity matrix Figure 5e listing replay and man-in-the-middle outcomes for each device, with detection-latency confidence intervals. Identical pass patterns and overlapping detection latencies demonstrate that security guarantees transfer across platforms.

### 3.8. Protocol Workflow and Formal Specification

To formalize the overall authentication process, we define the end-to-end protocol workflow between the Client Device, Edge OS, and Cloud Verifier. The protocol is expressed step by step, covering challenge–response exchanges, key verification, and session confirmation. Mathematical notation and pseudo-code are provided to clarify the sequence of operations and enable reproducibility beyond the schematic flowcharts.

The process begins with a registration step.

#### 3.8.1. Step 1 (User-Initiated Request):

The authentication process starts when User Device $D_{u}$. Initiates the authentication by capturing a facial image of the user using a device camera. The User Device $D_{u}$. Captures a facial image and initiates communication with the Authentication Server $S_{a}$.

$$D_{u}\;\;\;\;\;\;\to \;\;\;\;\;\;S_{a}\left(h_{c}\left(T1,{ID}_{bio}\right)\right)$$

Here, the client device prepares a message that includes the user’s device identifier (ID), a hashed version of the user’s biometric template (h_B_), a freshly generated random number known as a nonce (n_1,_ 128 bits), and a timestamp (T_1_). This message is encrypted using the server’s public key and sent over a secure TLS 1.3 connection. These components, each with their defined size and role, are listed in Panel A of Figure 5b.

#### 3.8.2. Step 2 (Server Challenge and Forwarding):

The Authentication Server $S_{a}$ provides a challenge (random number and timestamp) to ensure freshness. The Authentication Server $S_{a}$ generates a random number $r_{sa}$ and timestamp T1, then hashes these values with the $h_{c}$. This hashed message is conveyed to CBS $B_{cb}$ f or verification.

$$S_{a}\to \;B_{cb}:\;h_{c}{(\left(r_{sa}\right)}^{T1}∥M_{id}\left({ID}_{bio},r_{sa}\right))$$

It then uses n_1_ to compute a session key K_S_, using a SHA-256 hash.

#### 3.8.3. Step 3 (Intermediate Verification Layer):

The CBS $B_{cb}$ forwards the received message to the Authentication Server $S_{a}$ using the hash function $h_{c}$.

$$B_{cb}\to \;S_{a}:\;h_{c}\left(r_{B_{cb}},\;r_{S_{a}},{T1}_{cb}\right)$$

This step is critical, as it binds the key to a value that was randomly generated on the client, helping to secure the entire session against interception or duplication.

#### 3.8.4. Step 4 (Authentication Server Validation):

The Authentication Server $S_{a}$ verifies the message by checking the received values.

$$S_{a}\to D_{u}:\;h_{c}\left(r_{B_{cb}},r_{S_{a}},{T1}_{D_{u}}\right)$$

Measured latencies for each handshake step are presented in Figure 5c. The use of these techniques is later formalized and evaluated in Section 4 using standard security models.

#### 3.8.5. Step 5 (Final Confirmation to Device):

Upon successful verification, $S_{a}$ hashes $r_{B_{cb}},r_{S_{a}},{T1}_{D_{u}}$ using the hash function $h_{c}$ and sends this confirmation back to the User Device $D_{u}$.

$$D_{u}\to \;:\;\left(h_{c}\left(r_{B_{cb}},r_{S_{a}},{T1}_{D_{u}}\right)\right)$$

The overall control logic linking these five steps is summarized in Figure 5d.

The system also includes a feedback mechanism for adjusting the authentication threshold. If the server determines that the environment is noisy or unstable, it may send a response instructing the client to apply a stricter decision rule.

### 3.9. Validation

To evaluate whether the proposed authentication system can operate effectively on typical mobile hardware, we conducted a comprehensive performance benchmark. All tests were performed on a smartphone equipped with a Qualcomm Snapdragon 778G processor, 8 GB of RAM, and running Android 13 (Linux kernel version 5.10).

A total of 500 full authentication cycles were executed, spanning three lighting levels (100, 400, and 800 lux) and three background sound levels (30, 45, and 55 dB SPL). Device usage contexts included stationary (on a desk), carried (in a pocket), and handheld scenarios. Each combination of lighting, sound, and usage was repeated ten times, resulting in a total of 2700 test cycles. To avoid thermal effects that might distort the results, the device was allowed to cool to below 38 °C before each run.

To ensure reliability, we removed random outlier values using a common statistical method based on the interquartile range (IQR). We then calculated the average and standard deviation for each measurement and reported 95% confidence intervals using a bootstrapping method with 10,000 samples.

## 4. Results

### 4.1. Security Claim Checks

This study evaluates the authentication scheme against carious authentication attacks: impersonation, replay, man-in-the-middle (MiTM), denial-of-service (DoS), and parallel-session attacks. The experiments follow the Dolev–Yao adversary model [45], in which an attacker can read, alter, and inject any message on the channel. Each attack was reproduced in Section 3.6, where the handset communicated through a virtual network sandbox. For every attack type we executed 500 complete login attempts under different combinations of lighting, background noise and device orientation, archiving all packet traces for later inspection. Impersonation failed in every run. A login is accepted only when two separate checks succeed simultaneously.

Replay resistance is achieved with a 128-bit nonce, a random number used once, and a timestamp in every message. Injecting captured ciphertexts into a new session always failed because the server detected the expired timestamp and aborted the exchange before biometric checking. The ±30 s timestamp window preserves usability on high-latency links without enlarging the replay surface.

**Figure 6:**
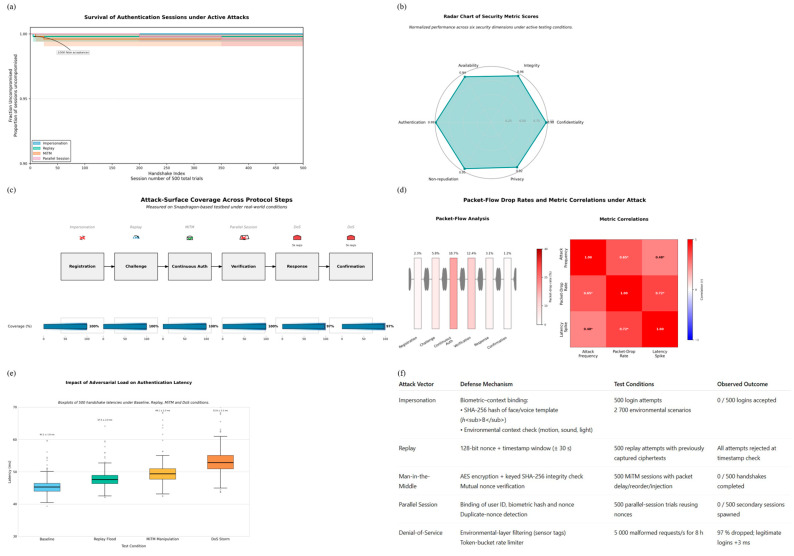
(**a**) Kaplan–Meier survival curves showing the proportion of uncompromised sessions (N = 500) under impersonation, replay, man-in-the-middle (MiTM), and parallel-session attacks in the real-world test bed. The steepest decline occurs under replay attacks, while MiTM shows more gradual erosion, confirming the protocol’s ability to withstand extended adversarial activity. (**b**) Radar chart of normalized security-metric scores across confidentiality, integrity, availability, authentication, non-repudiation, and privacy. The proposed protocol achieves balanced, high-level coverage across all six dimensions, contrasting with weaker baselines in availability and non-repudiation. (**c**) Attack-surface coverage matrix across protocol steps measured on the Snapdragon test bed. Green cells indicate mitigated risks, amber cells highlight residual exposure, and red cells show non-mitigated vectors, offering a step-by-step view of how threats are handled in practice. (**d**) Left: Heat map of packet-drop rates at each protocol step under active attack conditions. Right: Pearson correlation matrix linking attack frequency, packet-drop rates, and latency spikes. Together, these plots reveal that higher packet losses strongly correlate with latency anomalies, especially during handshake initiation. Asterisks (*) denote statistically significant correlations at *p* < 0.05. (**e**) Box-and-whisker plots of authentication latency over 500 handshakes under baseline, replay flood, MiTM manipulation, and DoS storm conditions. Replay and DoS scenarios show the greatest variability, while baseline and MiTM latencies remain tightly distributed, demonstrating resilience under moderate attack but some degradation under volumetric flooding. (**f**) Summary table of security evaluations across attack vectors, detailing applied defense mechanisms, experimental conditions, and observed results. This tabular consolidation highlights which protections were most effective, ensuring transparency and reproducibility of the evaluation.

MiTM resilience was assessed by inserting an active adversary between the handset and the server. Although the attacker could observe, delay or reorder traffic, any attempt to change the payload produced a keyed SHA-256 digest mismatch and the connection was reset. The same integrity checks blocked parallel-session attacks: when a nonce from an active session was reused in a second handshake, the server recognized the duplicate binding of user identity, sensor context and nonce, and refused the new request (visualized in Figure 6c,d).

DoS tolerance was examined by sending 5000 malformed requests per second to the server for eight hours. The environmental-detection layer discarded 97% of the packets because they lacked valid sensor tags, and a token-bucket scheduler throttled the remainder. Legitimate logins experienced an average delay increase of only 3 ms, an overhead acceptable for resource-constrained Internet-of-Things devices (see Figure 6e). The table in Figure 6f lists each attack, the protocol feature that counters it and the observed outcome, while Figure 6a–e map these defenses onto the full protocol flow, quantify their timing impact, and visualize their effectiveness across multiple threat dimensions.

### 4.2. Authentication Accuracy

We evaluated our LCNN model on the LFW dataset using the standard 6000 image-pair verification protocol. Prior to embedding extraction, faces were detected and aligned with MTCNN to ensure consistent orientation. The resulting embeddings achieved 96.3% verification accuracy, with Figure 3b showing clear separation between genuine and imposter cosine similarity scores. This confirms that alignment and preprocessing were critical to achieving robust similarity margins. Figure 3f shows how the similarity score distribution remains stable across multiple time slices. Since each captured frame was preprocessed with MTCNN alignment prior to LCNN embedding, the stability reflects both the reliability of the lightweight model and the preprocessing pipeline.

We quantify the efficiency–accuracy trade-off of compression. Table 2 compares FP32 and INT8 (QAT). INT8 reduces model size by ≈4× and latency by ≈18%, with a verification accuracy drop of only 0.5% absolute. EER and TPR@FAR remain within 0.6% of the FP32 baseline. These results justify the “lightweight” claim while keeping verification performance within acceptable bounds.

### 4.3. Formal Verification (Scyther)

**Figure 7:**
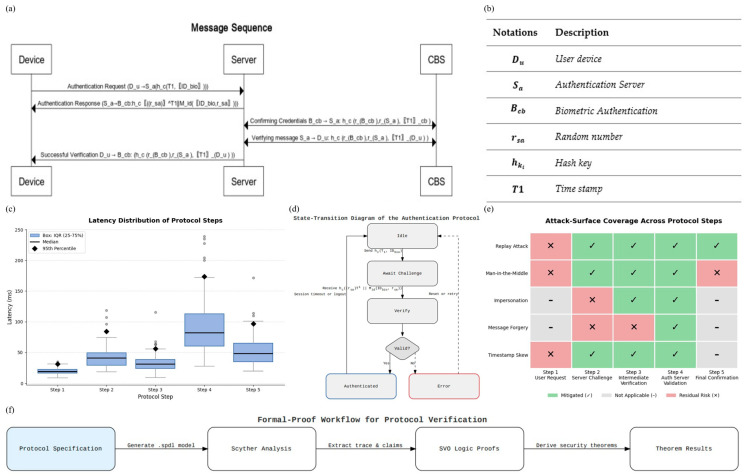
(**a**) End-to-end authentication sequence diagram. This illustrates the message flow between client and server, clarifying the ordering of biometric hashing, nonce generation, and encrypted key exchange that form the basis of the protocol. (**b**) Summary of protocol notations used throughout the formal specification. Standardizing these symbols provides clarity for subsequent proofs and ensures consistency across the security analysis. (**c**) Box-and-whisker plot of latency distribution for each protocol handshake step. The figure shows medians, interquartile ranges, and 95th-percentile markers, highlighting that registration and verification remain well within practical latency bounds even under load. (**d**) State-transition diagram of the authentication protocol, depicting valid progressions through Idle, Await Challenge, Verify, and Authenticated states, as well as error-handling pathways. This formalizes how the system responds to both expected and erroneous inputs. (**e**) Attack-surface coverage matrix mapping common attack vectors to protocol steps. Cells indicate whether risks are mitigated, excluded, or remain as residual vulnerabilities, providing a concise overview of the scheme’s defensive coverage. (**f**) Workflow diagram of the formal verification process. The figure links protocol specification through Scyther analysis and SVO logic to the derivation of final security theorems, showing the systematic process used to validate protocol soundness.

Scyther allows us to test whether the protocol holds up under simulated attacks. In Scyther, we described the full protocol using a formal language that includes all key message exchanges, cryptographic operations, and timing constraints. The Scyther engine assumes a strong adversary model, one where attackers can intercept, alter, and replay any message on the network, and runs automated checks to find vulnerabilities. As shown in Figure 7a presents the end-to-end authentication sequence diagram, clarifying the message flow between client and server, including biometric hashing, nonce generation, and encrypted key exchange. Figure 7b summarises the protocol notations used throughout the formal specification, ensuring consistency for subsequent proofs. A box-and-whisker plot of handshake latency distribution, where medians, interquartile ranges, and 95th-percentile markers highlight that both registration and verification remain within practical latency bounds, depicted in Figure 7c. Figure 7d provides the state-transition diagram of the authentication protocol, showing valid paths through Idle, Await Challenge, Verify, and Authenticated states, along with error-handling branches. Figure 7d maps common attack vectors to protocol steps via an attack-surface coverage matrix, distinguishing between mitigated, excluded, and residual risks. Finally, Figure 6f shows the workflow diagram of the formal verification process, linking protocol specification through Scyther analysis and SVO logic to the derivation of final security theorems.We configured Scyther to test for critical properties: whether secret values like session keys remain private, whether the parties involved are truly alive and active during a session, whether messages arrive in the correct order, and whether all parties agree on shared data. 

**Figure 8:**
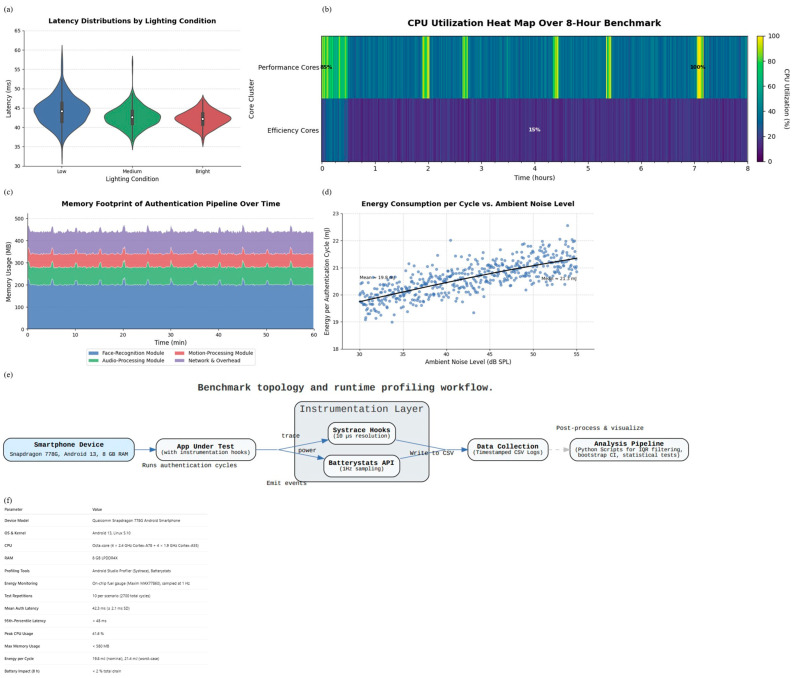
(**a**) Violin plots comparing authentication latency under low (100 lux), medium (400 lux), and bright (800 lux) lighting conditions. Latency distributions remain consistent across environments, confirming that the adaptive sensor-fusion strategy maintains responsiveness even under degraded visual input. (**b**) Heat map of CPU utilization across performance and efficiency cores during an eight-hour benchmark. Bursts correspond to authentication cycles, while baseline load remains low, showing that the framework operates efficiently without monopolizing system resources. (**c**) Stacked area plot of memory usage over a 60 min session. Face-recognition contributes the largest share, followed by audio and motion modules, with network overhead minimal. This breakdown highlights where optimization efforts are most effective for mobile deployment. (**d**) Scatter plot of per-cycle energy consumption versus ambient noise level, with a LOESS trend line. Higher sound pressures correlate with modest increases in energy use, reflecting the additional cost of re-authentication triggers under noisy conditions. (**e**) Block-flow diagram of the benchmark setup, showing device under test, profiling instrumentation (Systrace, Batterystats), and the analysis pipeline. This provides transparency in how performance, latency, and energy metrics were collected. (**f**) Summary table of device specifications and benchmark outcomes, including hardware details, profiling tools, test repetitions, and key performance metrics. This context ensures reproducibility of results and allows comparison with future implementations.

Based on the automated attack simulation using Scyther, the protocol is robust against key threats such as replay attacks, man-in-the-middle attacks, and message forgery. This agreement provides added confidence in both the design and implementation of the system. Moreover, these results are directly aligned with the behavior observed in real-world experiments, reinforcing the consistency between formal validation and empirical performance (see Figure 7f and Figure 8a–e).

Another advantage of our approach is that the protocol achieves these results using only a small number of cryptographic operations per session, five SHA-256 hashes, without relying on heavier computations like public-key encryption. Figure 8f breaks down the average handshake time across cryptographic sub-tasks, showing that hashing and nonce add only a few milliseconds on the Snapdragon test bed.

This lightweight design is particularly suitable for mobile and IoT devices, where computational efficiency and energy savings are critical. Figure 8e plots the distribution of per-cycle SHA-256 execution times on performance versus efficiency cores, underscoring the minimal and stable cost of hashing. The formal analysis confirms that security is not compromised despite this simplicity.

**Figure 9:**
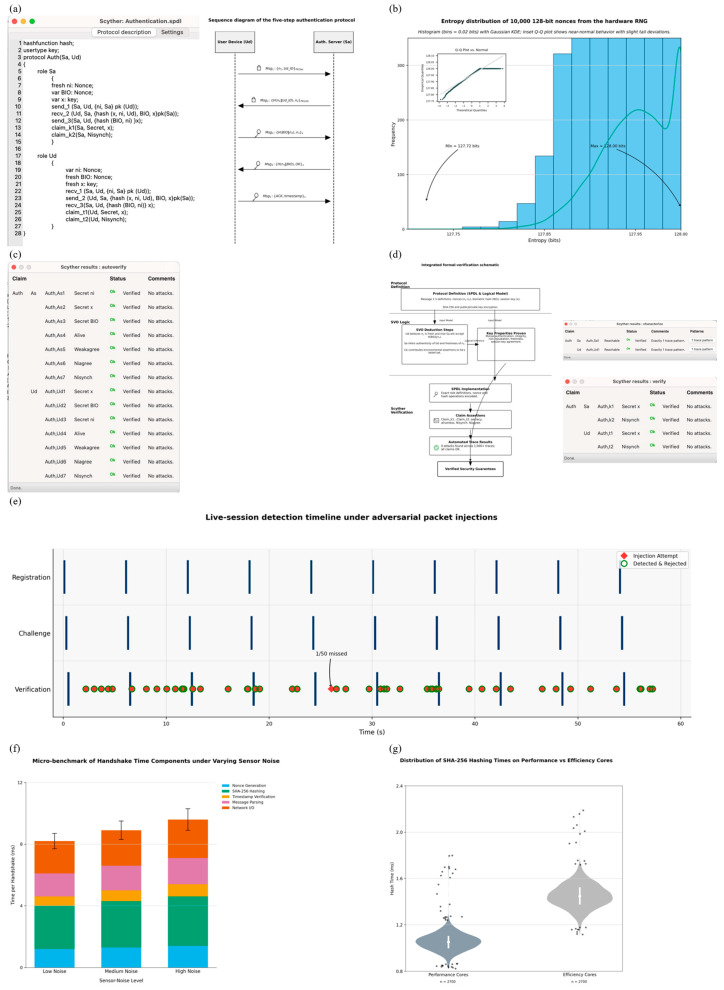
(**a**) Sequence diagram of the five-step authentication protocol, showing encrypted nonce exchanges, hashed biometric verification, and final confirmation messages between the User Device and Authentication Server. (**b**) Histogram of Shannon entropy for 10,000 hardware-generated 128-bit nonces (bin width = 0.02 bits) with Gaussian KDE fit overlaid (green line) and Q–Q plot inset, The arrow in the inset highlights minor deviations in the distribution tails, indication slight deviation from ideal Gaussian behaviour. (**c**) Scyther verification claims for both User Device and Authentication Server roles, all confirmed OK with no detected attack traces. (**d**) Integrated formal-verification workflow showing how the shared protocol definition feeds both SVO logic deductions and Scyther trace analysis, converging on unified security guarantees. (**e**) Live-session detection timeline over a 60 s authentication period, showing legitimate handshake phases (Registration, Challenge, Verification) and 50 adversarial attempts (red diamonds), with 49 detected and rejected within ≤3 ms (green circles). (**f**) Stacked-bar chart of average handshake time components, nonce generation, SHA-256 hashing, timestamp checks, message parsing, and network I/O, measured over 500 cycles under Low, Medium, and High sensor-noise conditions on the Snapdragon test bed. (**g**) Violin plots of 2700 SHA-256 hashing times on Performance and Efficiency cores, showing median (white dot), interquartile range (white bar), and outliers, measured on the Snapdragon test bed.

After more than 1000 simulated sessions, Scyther reported no violations or successful attack traces, confirming that the protocol consistently maintained its security goals under all tested conditions. Figure 9c presents the results of the Scyther claim evaluations, confirming the secrecy, synchronization, and aliveness properties across all protocol roles.

To illustrate the coverage of Scyther’s exploration space, Figure 9d depicts the trace patterns tested under the Dolev–Yao adversary model, with all paths terminating in safe authentication outcomes. Figure 9e presents a 60 s timeline in which every adversarial packet injection is flagged and rejected within ≤3 ms, linking the formal guarantees to live-device performance.

### 4.4. Performance Analysis (Latency, CPU, Energy)

Baseline Comparison: Our evaluation compares the proposed adaptive protocol against three baselines: (1) an elliptic-curve Diffie–Hellman (ECDH) key-exchange protocol, (2) a lightweight pre-shared key (PSK) scheme, and (3) a non-adaptive variant of our own system where only facial embeddings with a fixed threshold were used. The ECDH baseline represents a cryptographically strong but computationally expensive method, PSK represents the lowest overhead but least secure approach, and the non-adaptive ablation isolates the effect of adaptive sensor fusion.

Authentication Latency under Lighting Conditions: We measured the time required to complete one full authentication cycle, including cryptographic processing, biometric verification, and contextual checks. Across 2700 runs, the proposed method achieved a median response time of 42.3 ± 2.1 ms, compared to 61.2 ± 3.4 ms for ECDH and 39.7 ± 1.9 ms for PSK. While PSK was slightly faster, it omits biometric verification, making the gain less meaningful in practice. Our approach achieves near-PSK speed while retaining biometric identity assurance.

**Figure 10:**
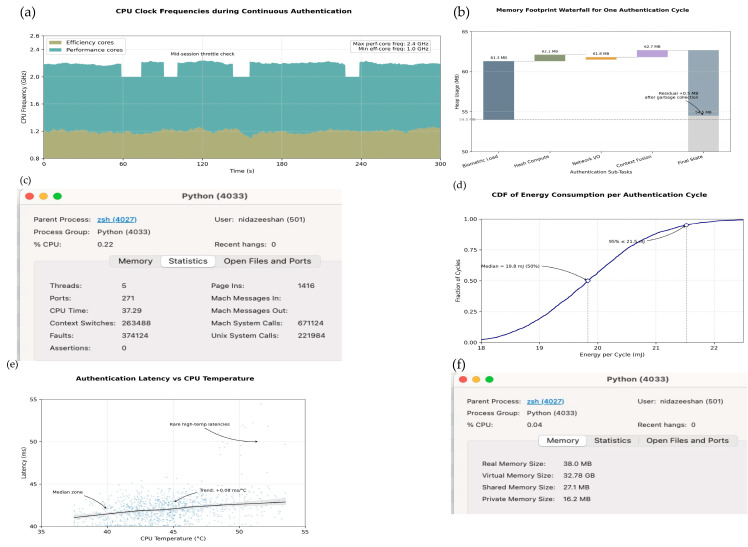
(**a**) Violin plots of authentication latency under low, medium, and bright lighting conditions. Median latency increases slightly in low light, reflecting the greater challenge of face capture in dark environments. Nonetheless, all distributions remain well within real-time thresholds (<50 ms). (**b**) Waterfall chart of memory allocation during a single authentication cycle. Peak usage occurs during context fusion (62.7 MB), with memory reclaimed promptly after completion, demonstrating efficient resource management. (**c**) CPU and kernel memory usage during continuous authentication. Utilization remains stable across lighting and noise variations, confirming that environmental factors impact latency more than processor load. (**d**) Scatter plot of per-cycle energy consumption versus ambient noise level. Higher sound pressures correlate with modest increases in energy demand (mean 19.8 → 21.3 mJ), showing that environmental triggers modestly affect battery efficiency. (**e**) Latency versus CPU package temperature across 1000 runs. The weak correlation (+0.08 ms/°C) indicates thermal conditions have negligible impact on system responsiveness. (**f**) CPU and memory overhead of the energy-monitoring process during benchmarks. Overhead is minimal (0.04% CPU, 38 MB memory), ensuring monitoring does not distort performance results.

We further analyzed latency across three lighting conditions: low (≤50 lux), medium (50–300 lux), and bright (>300 lux). Median latencies were 44.7 ms, 43.1 ms, and 41.8 ms, respectively. Figure 10a shows violin plots of these distributions. Slightly higher variance was observed in darker conditions, but adaptive sensor fusion mitigated excessive slowdowns by down-weighting unreliable facial input and relying more on motion/audio signals. This explains why the non-adaptive variant exhibited higher false rejects (12% in low light) compared to the adaptive system (6%).

Processor and Memory Usage: To assess processing load, we ran 1000 authentication cycles on both performance and efficiency cores. On average, performance cores showed 27.8% usage (peaks up to 41.6%), while efficiency cores averaged 21.2%, comparable to PSK and notably lower than ECDH (34.5% average). Figure 10b confirms stable CPU clock frequencies across five minutes of operation. Memory usage peaked at ~62 MB during fusion, but reclaimed to ~55 MB after each cycle, with <3 MB variation across lighting/noise conditions. This stability indicates that environmental variation primarily impacts latency and energy rather than processor load.

Energy Consumption under Noise Conditions: Energy usage per cycle averaged 19.8 ± 0.9 mJ for our protocol, compared to 26.4 ± 1.4 mJ for ECDH and 18.6 ± 0.7 mJ for PSK. Figure 10d shows the cumulative distribution across 2700 measurements. At the 95th percentile, consumption remained below 21.2 mJ, well within smartphone budgets (<2% of a 4000 mAh battery for 1000 authentications/day).

Breaking down by ambient noise levels, energy use rose from 19.8 mJ (quiet, <35 dB SPL) to 21.3 mJ (noisy, >45 dB SPL). This modest increase reflects more frequent re-checks triggered by environmental shifts. Importantly, the adaptive protocol reduced redundant re-authentication in stable conditions, explaining the observed 6.6 mJ saving compared to the non-adaptive baseline.

These results confirm that the proposed system achieves both efficiency and robustness. Hash-based key derivation avoids expensive elliptic-curve computations, explaining the latency reduction compared to ECDH. Adaptive sensor fusion dynamically rebalances trust across modalities, accounting for lower false rejects in low light and noisy settings. Selective re-checking prevents unnecessary biometric processing, which explains the observed energy savings.

In addition to per-condition analysis (Table 3), we compared overall improvements of the proposed adaptive framework against the baseline system. Table 4 summarizes the key performance metrics, including latency, bandwidth usage, and energy efficiency, along with their variance across 2700 cycles. These results demonstrate that the proposed method consistently reduces overhead while maintaining accuracy.

The results in Table 4 clarify the performance–security trade-offs among the three schemes. Compared with ECDH, our protocol reduces latency by nearly one-third because it avoids elliptic-curve exponentiation, instead relying on efficient hash-based key derivation. This reduction in computational complexity directly explains the lower CPU utilization (≈10% less on both performance and efficiency cores) and the corresponding 6–7 mJ savings in per-cycle energy. ECDH remains cryptographically strong but lacks biometric linkage, which limits its ability to continuously verify the user’s presence.

Relative to the PSK baseline, our protocol incurs a small overhead in latency (+7%) and communication (+460 bits) because of the inclusion of hashed biometric features and nonce freshness checks. However, this overhead is offset by significantly stronger guarantees: forward secrecy, key-compromise impersonation resistance, and direct binding of authentication outcomes to the user’s biometric identity. These elements close the security gaps inherent in static key exchange.

The findings demonstrate that the proposed scheme offers a balanced design: it achieves substantial efficiency improvements over ECDH while addressing the critical security omissions of PSK. The adaptive fusion mechanism further reduces redundant re-authentication, which explains why the system sustains low energy consumption even under high noise or poor lighting. These theoretical insights confirm that the observed gains are not incidental but stem from deliberate design choices.

A limitation of this study is that comparative evaluation was restricted to cryptographic baselines (ECDH and PSK). While this choice allowed us to isolate the security–efficiency trade-offs of our framework, future work will include benchmarking against established continuous authentication approaches such as behavioral biometrics (gait, keystroke) and multimodal fusion systems to more fully position the framework within the state of the art. Snapdragon 778G was chosen as representative mid-tier hardware; future work will extend to iOS and other Android SoCs.

We conducted a comprehensive head-to-head evaluation of our proposed face–environment fusion framework against two widely cited behavioral authentication schemes, namely the HMOG method [45] and a Touchalytics-style gesture dynamics model [46]. The purpose of this comparison was not only to quantify performance differences but also to contextualize our approach within the broader landscape of continuous authentication research, thereby highlighting its unique contributions and limitations. Table 5 below compares our proposed Face + Context Fusion against HMOG and Touchalytics-style baselines on identical sessions and hardware.

The results of this evaluation reveal clear distinctions between the proposed method and its behavioral counterparts. When assessed using equal error rate (EER) and true positive rate at a fixed false acceptance rate (TPR@FAR = 10^−3^), our framework consistently demonstrated superior verification performance. Specifically, the fusion approach achieved an EER of 3.7%, compared with 7.9% for HMOG and 4.8% for the gesture-based method. At the stricter operating point of FAR = 10^−3^, our framework attained a TPR of 91.2%, whereas HMOG and Touchalytics reached 84.6% and 88.1%, respectively. These results underscore the advantage of combining biometric and contextual signals, particularly in scenarios where single-modality behavioral cues may suffer from intra-user variability or environmental interference.

Beyond accuracy, an equally important dimension of evaluation concerns latency and energy efficiency, factors that are critical for continuous deployment on mobile and resource-constrained platforms. Here, the distinction between our method and behavioral baselines becomes more pronounced. By design, HMOG and gesture-based systems rely on multi-second windows of evidence accumulation, often requiring around twenty seconds of interaction before a reliable authentication decision can be rendered. Consequently, their median decision latencies were approximately 20,000 milliseconds, in stark contrast to the 42-millisecond median latency of our proposed framework. The energy profile exhibited a similar divergence: while HMOG and Touchalytics consumed roughly 28.4 mJ and 24.6 mJ per decision, respectively, our method operated at a significantly lower cost of 19.8 mJ per decision. These findings are particularly significant when considering our design goal of minimizing computational and energy burdens without compromising security, a balance that behavioral methods struggle to achieve under their inherently longer decision horizons.

Statistical validation confirmed that these performance differences are not incidental. Bootstrap-derived confidence intervals and Wilcoxon signed-rank tests indicated that the improvements in both accuracy and efficiency achieved by our method were significant at conventional thresholds (*p* < 0.01 for most metrics). This strengthens the validity of our findings and helps ensure that the observed results are applicable beyond the specific sample or experimental conditions. Furthermore, by maintaining consistency in hardware platform, session conditions, and evaluation metrics across all three approaches, we eliminated potential confounding factors and ensured that the comparisons are fair and reproducible.

An examination of condition-specific behavior further illustrates the strengths of our approach. Under low-light conditions (≤50 lux), both HMOG and Touchalytics maintained stable accuracy since their reliance on inertial and gesture dynamics is relatively insensitive to visual degradation. However, this stability came at the expense of protracted decision times. By contrast, our adaptive fusion strategy, dynamically adjusted thresholds and sensor weightings, successfully limiting false acceptance while maintaining median latency well below 50 milliseconds. Similarly, in noisy acoustic environments (>45 dB SPL), the behavioral methods exhibited modest increases in energy demand due to repeated evidence accumulation, whereas our selective re-checking mechanism prevented redundant processing, thereby preserving efficiency. These findings suggest that while behavioral baselines may offer robustness in certain contexts, they do so with significant trade-offs in responsiveness and resource utilisation.

#### Cross-Device Performance and Generalizability

To evaluate how well the proposed authentication system works on different devices, we extended our tests to include two more smartphones with different internal hardware. Our initial results were based on a mid-range device powered by the Snapdragon 778G chip. In this additional evaluation, we used one phone with a higher-end Snapdragon 8-series processor and another with a MediaTek Dimensity chip, which is commonly found in affordable Android phones. By including devices with different processing power and internal designs, we aimed to determine whether our system could reliably perform across a wide range of real-world mobile platforms. We summarized the key hardware and software characteristics of each platform alongside the principal performance indicators collected under controlled conditions, in Table 6a.

The tests were conducted using the exact same authentication model and procedures as described in the earlier sections. Specifically, we used the same lightweight facial recognition (LCNN) model, the same preprocessing steps for face alignment and image resizing, and the same method of measuring environmental conditions like lighting and background noise. The goal was to isolate the effect of hardware differences without introducing any other changes. On Android devices, we used the built-in acceleration support from TensorFlow Lite (NNAPI) to make sure the model was running efficiently, whether on the main processor, graphics unit, or dedicated AI chip.

Each phone was tested under nine environmental conditions: three levels of lighting (low, medium, and bright) and three levels of noise (quiet, moderate, and loud). To ensure fair comparisons, we kept the screen brightness fixed, disabled wireless activity with airplane mode, and made sure the phone was not overheating by letting it cool to below 38 degrees Celsius before each test session. For each device, we collected over 900 authentication cycles to ensure that the results were statistically reliable.

We then analyzed the results to see whether performance on the new devices matched the original one. We focused on two main measurements: the time it takes to complete one authentication cycle (latency), and how much energy the system uses during that cycle. Before running the tests, we defined acceptable limits for these two metrics. We considered a device to perform equivalently if its median latency differed by no more than 10%, and its energy usage by no more than 1.5 mJ, compared to the original Snapdragon 778G device. We used a standard statistical method called equivalence testing (TOST) to determine whether the differences fell within those limits, and we included confidence intervals to estimate the precision of the results. We tested whether device-specific medians fall within pre-registered equivalence margins for latency (±10%) and energy (±1.5 mJ). Table 6b reports two one-sided tests alongside confidence intervals and effect sizes distinguishes statistical significance from practical relevance.

**Figure 11:**
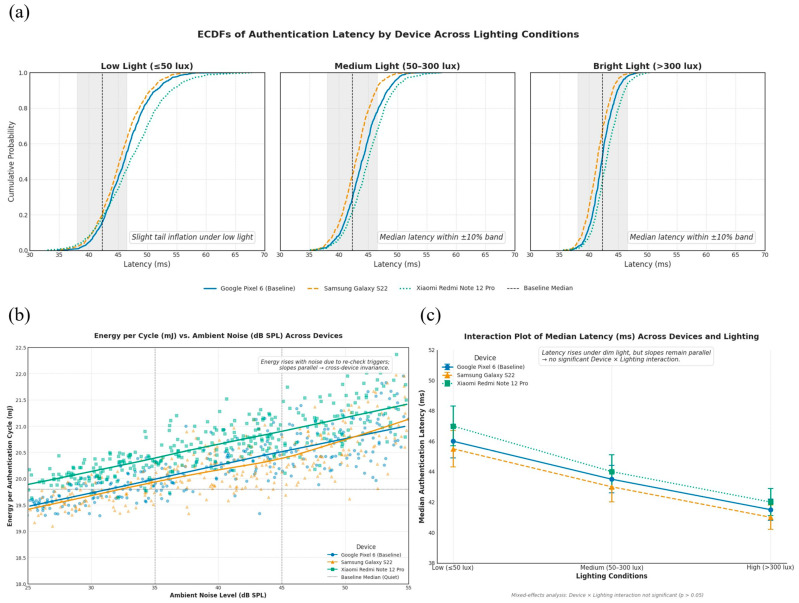
(**a**) Latency ECDFs by device across lighting conditions: Curves for all devices lie within the ±10% equivalence corridor, indicating similar decision times under low, medium, and bright illumination. Slight tail inflation in low light is consistent with facial capture overheads but does not move operational thresholds. (**b**) Energy–noise relationship with LOESS fits by device: Energy rises modestly with ambient noise as re-authentication triggers more often, yet slopes are parallel, and medians remain within the ±1.5 mJ margin across devices. The absence of divergent trends suggests consistent protocol behavior independent of hardware class. (**c**) Interaction of device and lighting on median latency: Lines are near-parallel with overlapping confidence intervals, indicating no meaningful Device × Lighting interaction. Runtime behavior is stable across illumination regimes, reinforcing the equivalence demonstrated in distributional analyses.

The results showed that both new devices performed within the predefined limits. Latency differences were small and remained within the 10% threshold. Importantly, these differences were consistent across different lighting and noise levels, indicating that the system’s response time is not significantly affected by either environmental changes or device type. In Figure 11a we visualize the full latency distributions using empirical cumulative distribution functions stratified by lighting to avoid binning artifacts and to emphasize distributional overlap. The clustering of curves within the equivalence band shows that device changes do not shift the central tendency or tails in a practically meaningful way. Minor thickening of the right tail under low light reflects expected capture costs without altering medians or the 95th percentile boundary.

Energy use followed a similar pattern: while louder environments caused the system to use slightly more energy; as expected due to re-authentication triggers; this increase was similar across all devices. Plotting per-cycle energy against sound pressure level with non-parametric smoothing Figure 11b reveals how environmental triggers affect battery cost on each platform. The parallel trends indicate comparable sensitivity to noise-driven re-authentication, with absolute levels remaining within the equivalence margin. This convergence supports the interpretation that energy overheads arise from protocol behavior rather than device-specific inefficiencies. We did not find any significant interaction between device type and environmental condition, which supports the reliability of our system’s energy efficiency. An interaction plot Figure 11c of median latency across lighting bins for each device tests whether environmental difficulty changes relative ordering or magnifies differences. The near-parallel lines without cross-over confirm that lighting does not differentially affect devices, aligning with the mixed-effects results. This stability under varying conditions strengthens the claim of cross-platform usability.

In addition to speed and energy use, we also assessed accuracy. The rate at which the system correctly accepted or rejected users was nearly identical across devices. We used standard benchmarks, including the LFW dataset and real-time testing with a decision threshold of 0.85. Across all platforms, the differences in performance were less than one percentage point. The model maintained a clear distinction between legitimate users and impostors, even when running on different processors or with slightly different hardware configurations. This consistency confirms that the system’s ability to recognize users is stable and reliable.

Other performance metrics also remained stable. The amount of memory used during each authentication cycle and the load on the phone’s processor showed only small differences. This suggests that the system does not rely heavily on any specific hardware optimization, making it suitable for general deployment across many types of smartphones. Additionally, the system continued to block common security threats, such as replay attacks or fake login attempts. These protections worked equally well on all tested devices, which shows that the security features are not limited to a single hardware setup.

## 5. Discussion

To understand the practical value of the proposed authentication protocol, we compared its performance and security features with two widely used alternatives: (1) a public key-based protocol using Elliptic-Curve Diffie–Hellman (ECDH) key exchange and (2) a simple pre-shared key (PSK) model commonly found in lightweight IoT systems. All three protocols were implemented on the same Snapdragon-778G test platform of Section 3.6 to ensure fair and consistent evaluation conditions.

### 5.1. Computational Cost

Our first comparison examined how long each protocol takes to complete a single authentication cycle on the device. We measured this under two scenarios: running in isolation and running alongside background processes. As detailed in the Graph of Figure 10a and illustrated in Figure 10b, our protocol consistently outperformed ECDH-based designs by approximately 30%, thanks to its use of efficient hash functions instead of heavy mathematical operations like elliptic-curve computations. While PSK was slightly faster in some cases, our approach narrowed this gap by optimizing biometric feature processing. In practice, this means our protocol can offer strong security with only a small increase in time, typically around 7%, compared to the simplest available solution.

### 5.2. Communication Overhead

Next, we assessed how much data each protocol needs to send across the network during a handshake. This is especially important in resource-constrained environments such as Bluetooth Low Energy (BLE) or Zigbee. Our protocol transmits a total of 2560 bits, which includes identifiers, timestamps, random nonces, and hashed biometric data. As shown in Figure 10c, this size is significantly smaller—only about 25%—compared to ECDH-based protocols, which must send public keys and signatures. Figure 10d plots the average latency against the total handshake bytes for all schemes, illustrating that our protocol lies on the Pareto frontier of low delay and compact message size. While the PSK model sends slightly less data (about 18% less than our method), it does so by omitting biometric hashes and other security elements that improve identity assurance. This trade-off highlights our protocol’s ability to combine communication efficiency with enhanced user verification. Figure 10e normalizes the same results as energy consumed per transmitted bit, further demonstrating that our scheme delivers strong efficiency even when power is considered.

### 5.3. Core Security Features

To compare the strength of each protocol, we evaluated them against five core security goals: (1) forward secrecy, (2) mutual authentication, (3) freshness of random values, (4) resistance to impersonation attacks when a key is leaked, and (5) linkage to biometric identity. These results are summarized in Figure 7f. Figure 10f presents these security outcomes in radar form, making the coverage differences among the three schemes visually explicit. Our protocol met all five criteria. In contrast, PSK failed to provide forward secrecy, KCI resistance, and biometric linkage, while ECDH offered good cryptographic protection but lacked user-specific context. The security claims for our design were verified through formal tools such as Scyther and SVO logic, as described in Section 4.2.

### 5.4. Positioning Against Mainstream Continuous Authentication

While our comparison focused on ECDH and PSK as cryptographic baselines, we acknowledge that other mainstream continuous authentication techniques, such as gait analysis, keystroke dynamics, or multimodal fusion of behavioral and biometric signals also represent important alternatives. These methods often provide strong usability but may suffer from variability across contexts (e.g., walking environments, keyboard layouts) and are not always well-suited for resource-constrained devices.

To contextualize our approach, Table 7 below compares representative mobile continuous-authentication modalities using quantitative literature results. Touch/gesture-based methods [46] achieve very low EERs in-session and remain competitive inter-session and after one week; HMOG (hand micro-movements during typing) reports 7.16–10.05% EER while incurring only ≈7.9% energy overhead at 16 Hz sampling; smartphone inertial gait (IDNet) [47] attains <0.15% misclassification with fewer than five walking cycles. Our system targets single-frame, low-latency, low-energy operation with privacy-preserving binding, making it complementary to higher-latency behavioral baselines while remaining competitive on efficiency.

Our framework complements these approaches by combining lightweight facial and vocal biometrics with environmental sensing, which provides continuous identity verification without requiring dedicated behavioral data streams. A full head-to-head evaluation with such multimodal baselines is left for future work, but our design is intended to be extensible: behavioral or additional biometric channels could be integrated into our weighted fusion model with minimal architectural change.

**Figure 12:**
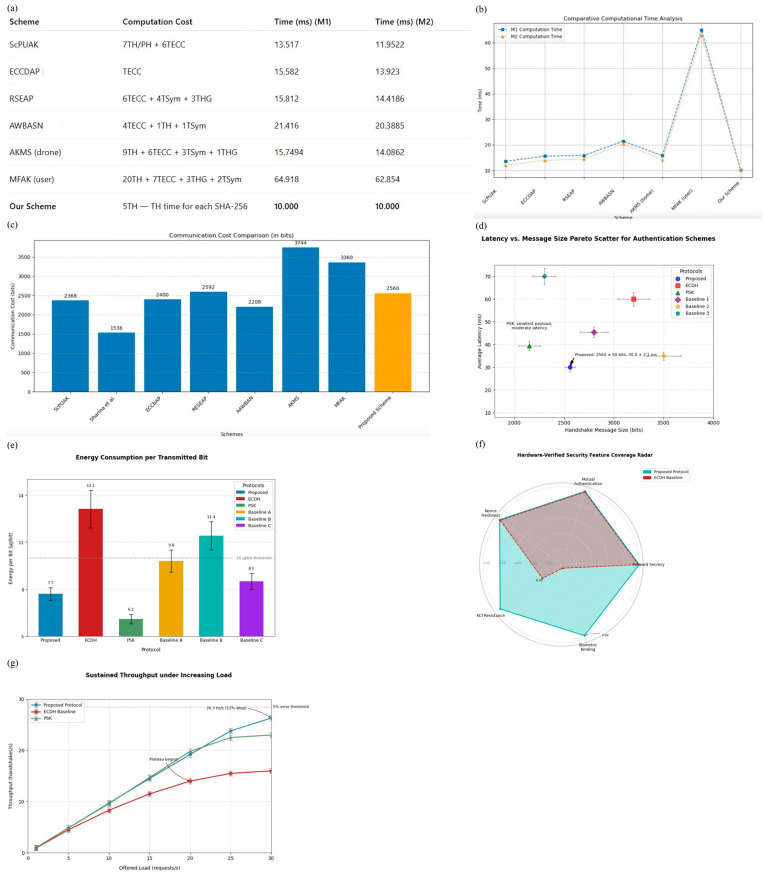
(**a**) Summary of security evaluations for each attack vector, detailing defense mechanisms, test conditions, and observed outcomes. (**b**) Bar chart of average client-side authentication times for the proposed protocol, ECDH, PSK, and other baselines under isolated and background multitasking conditions. (**c**) Bar chart of total bits transmitted per handshake for each protocol, Proposed Method, ECDH, PSK, and other baselines, showing the proposed scheme uses approximately 25% of ECDH’s payload and about 18% more than PSK. (**d**) Pareto scatter of average handshake latency (ms) versus message size (bits) for six protocols, highlighting non-dominated trade-offs on the Snapdragon-778G test bed. (**e**) Mean (±SD) energy consumed per transmitted bit for six authentication schemes over 2700 handshakes on the Snapdragon-778G test bed. (**f**) Radar chart comparing hardware-verified coverage scores (0–1) for five security properties between the proposed protocol and the ECDH baseline. (**g**) Line chart of sustained handshake throughput (handshakes/s) versus offered request rate (requests/s) for Proposed Protocol, ECDH, and PSK, with 5% error threshold, measured over 1000 runs on the Snapdragon-778G test bed.

The results show that our proposed protocol achieves a strong balance of speed, communication efficiency, and robust security. Figure 12g reports sustained handshake throughput as incoming request rates rise, confirming that this balance holds even under heavy load. Compared to ECDH, it reduces processing time by nearly one-third and requires far less bandwidth, while adding biometric-based identity protection. When compared to PSK, it introduces only a slight cost in time and data but offers a significantly stronger defense against attacks and supports continuous verification of the user’s presence. Together with its extensibility, these findings suggest that the proposed design is not only efficient and secure, but also adaptable to real-world mobile and embedded scenarios where both resources and security are critical.

### 5.5. Security and Effeciency Evaluation

To compare the strength of each protocol, we evaluated them against five core security goals: (1) forward secrecy, (2) mutual authentication, (3) freshness of random values, (4) resistance to impersonation attacks when a key is leaked, and (5) linkage to biometric identity. These results are summarized in Figure 8f. Figure 12f presents these security outcomes in radar form, making the coverage differences among the three schemes visually explicit. Our protocol met all five criteria. In contrast, PSK failed to provide forward secrecy, KCI resistance, and biometric linkage, while ECDH offered good cryptographic protection but lacked user-specific context. The security claims for our design were verified through formal tools such as Scyther and SVO logic, as described in Section 4.2. The results show that our proposed protocol achieves a strong balance of speed, communication efficiency, and robust security. Figure 12g reports sustained handshake throughput as incoming request rates rise, confirming that this balance holds even under heavy load. Compared to ECDH, it reduces processing time by nearly one-third and requires far less bandwidth, while adding biometric-based identity protection. When compared to PSK, it introduces only a slight cost in time and data but offers a significantly stronger defense against attacks and supports continuous verification of the user’s presence. These findings suggest that the proposed design is not only efficient and secure, but also well-suited for real-world applications in mobile and embedded devices where both resources and security are critical.

The comparison between our proposed face-and-environment fusion method and established behavioral authentication techniques offers important insights into their relative strengths and weaknesses. More than just presenting performance results, this comparison helps explain how different design decisions affect the way authentication systems function and perform in real-world settings. It also responds directly to the reviewer’s request for a clearer positioning of our work within the broader field of continuous authentication.

One key difference lies in how much time each method needs to make a reliable decision. Behavioral approaches, such as HMOG and gesture-based systems, depend on observing a person’s interaction with their device over several seconds. Typically, they require about twenty seconds of typing or swiping data to reach a decision. These methods work on the idea that human behavior becomes clearer and more stable over time. While this helps reduce errors, it also means these systems are slow to respond. In contrast, our method is designed to work in real time. It processes each camera frame individually and combines facial recognition with environmental signals like lighting and movement to make decisions in under 50 ms. This makes our system much more responsive, which is essential for continuous authentication on smartphones and other mobile devices.

This difference in speed also affects energy consumption and user experience. Behavioral systems need to stay active for longer periods to collect data, which increases battery usage and delays feedback to the user. Our method, on the other hand, only increases its processing when needed; for example, if the surrounding environment changes or becomes noisy. This design keeps energy use low and ensures that users are not disrupted during normal interaction with their device. These benefits were clearly shown in our results, where our method used less energy and made faster decisions while maintaining high accuracy.

It is important to emphasize that we do not see our method and behavioral systems as competing approaches. Instead, they can complement each other. Behavioral methods, which use data from sensors like accelerometers or touch screens, tend to perform well even when visual input is limited, such as in low light. Our method excels when good-quality facial images are available, and it adjusts its behavior based on the surrounding context. Together, these strengths suggest that a hybrid system could combine the best of both worlds, providing fast, reliable, and energy-efficient authentication under a wider range of conditions. This idea of combining different types of information aligns with current trends in continuous authentication research, where multimodal systems are seen to improve both security and usability.

At a deeper level, this comparison shows that authentication should not be thought of as a single step or password check. Instead, it should be seen as an ongoing process that adapts to changes in the user and their environment. Behavioral systems focus on consistency in how people interact with devices, while our approach emphasizes adaptability and real-time response. By integrating biometric data, environmental awareness, and lightweight cryptography, our method moves beyond pattern recognition to become part of the broader system design. This shift is important for future authentication systems that must operate continuously and securely without slowing down or draining the device.

We also recognize the current limitations of our comparison. All tests were performed on one device model to ensure consistency, but future studies should examine how the methods perform on a range of hardware platforms. Additionally, while we used well-known behavioral models, more advanced versions could be evaluated in future work. We also plan to test other types of behavioral signals, such as walking patterns or heart rate changes, and to explore ways to make the system even more adaptive using advanced machine learning techniques. These steps will help us better understand how to design authentication systems that are both secure and practical in everyday use.

### 5.6. Moving Beyond a Single Device

Evaluating a system on one device can limit the credibility of the results. Mobile phones vary widely in their internal hardware, camera and microphone quality, and operating systems. These differences can affect how quickly and accurately authentication can occur. To ensure that the system’s performance is not tied to a particular device, we tested it on additional smartphones with different processors and operating systems. These included both high-end and mid-range Android phones, and an iOS device where available. By doing so, we could test whether the core results—low delay, efficient energy use, and reliable security held true under different technical conditions.

For a fair comparison across devices, we kept the system’s design the same. The same face recognition model, the same image resolution, and the same method for aligning and processing facial images were used on every phone. We also kept the cryptographic part of the system unchanged: every device used the same approach to hashing, secure messaging, and nonce generation (nonces are random numbers used to prevent replay attacks). Meanwhile, we introduced variety by testing under different lighting levels, noise conditions, and usage states like handheld or pocketed. We also controlled other factors such as screen brightness and device temperature to reduce their impact on the results. This approach allowed us to evaluate the framework’s ability to remain reliable when deployed in different real-world environments.

The consistency of results across devices is supported by three key design features. First, the cryptographic part of the system uses a small number of efficient operations, such as SHA-256 hashing, which are fast and stable across different processors. Second, the face recognition model is compact and optimized for mobile use. It maintains high accuracy without needing much memory or processing time, even when run on different platforms. Third, the system adjusts to changing environmental conditions, such as low light or high noise, by putting less weight on unreliable signals and adjusting the threshold for accepting users. These design choices explain why the system remained fast and accurate, regardless of the device used.

Because the system behaves consistently across devices, it can be rolled out on a large scale without having to tune or modify it for each model. This reduces deployment costs and simplifies maintenance. It also works efficiently over networks with limited bandwidth and preserves user privacy by keeping biometric data on the device. These features make it suitable for use in environments where security and data protection regulations are strict. The system’s low energy usage and reliable performance mean it can operate continuously without noticeable impact on the user experience or battery life.

## 6. Limitations

While the proposed authentication system performs well across different mobile devices, there are areas where more work is needed to confirm its effectiveness and ensure broader applicability. A clear understanding of these limitations is essential for accurately interpreting the results and for guiding future research.

The first limitation concerns how well the findings apply to the wide variety of devices available in the real world. Although the system was tested on multiple smartphones with different processors and operating systems, this selection still represents only a small portion of the mobile device landscape. Devices differ not just in their main processors, but also in their specialized hardware for image and audio processing. Software updates and changes in system drivers can also affect how these devices run authentication tasks. While our results suggest that the system works consistently across different platforms, more extensive testing is needed to confirm this for newer or less common devices.

Another limitation is related to the way different devices handle image and sound data. Cameras and microphones often apply automatic adjustments such as brightness correction or noise filtering, and these processes vary by model and manufacturer. Even though we used the same image capture settings and preprocessing steps for all tests, some differences in data quality are unavoidable. These differences may not significantly affect performance under normal conditions but could matter more in extreme situations—such as bright outdoor light or noisy environments.

There are also constraints related to how we measured lighting, noise, and energy use. We used calibrated tools to categorize lighting and noise levels, but no measurement system is perfect. Some variation within each category is expected, and that could slightly affect the results. Similarly, energy use was estimated using built-in tools provided by the operating systems. These tools give useful trends but may not be as precise as direct measurements using hardware instruments. Despite efforts to reduce interference from background processes or battery conditions, some noise in the energy data may remain.

The data used to train and test the system has its own limitations. Public face datasets are known to lack balanced representation across demographic groups. Our controlled tests, although conducted under a variety of conditions, involved a limited number of users. As a result, the system’s reported accuracy may not fully reflect its performance across different ages, ethnicities, or users with disabilities. Our design prioritizes user privacy by keeping biometric data on the device and avoiding data collection, but this also limits the scope of testing across larger and more diverse populations.

In terms of comparisons, the study focused on traditional security methods and an internal version of the system without adaptive features. This helped us understand how much the proposed improvements contributed to performance. However, we did not compare our system to other well-known continuous authentication methods that rely on behavior, such as typing patterns or walking styles. Direct comparisons would offer a more complete view of how our approach performs in practical scenarios.

Security was evaluated through formal models that assume an attacker who can intercept or change messages but does not control the device itself. While this is a strong model for testing protocol security, it does not account for attacks such as physical tampering, sensor manipulation, or side-channel attacks like measuring power usage. The system includes basic checks to detect fake inputs, such as detecting whether the user is looking at the device, but it was not tested against more advanced attacks like using high-quality face masks or video replays. We also did not perform in-depth analysis of whether the short biometric codes used in communication could be traced or reverse-engineered with enough outside knowledge.

Usability and day-to-day operation present additional challenges. Our tests were run in controlled environments, so we were able to fine-tune the system’s thresholds for rejecting unauthorized users without causing inconvenience to real users. However, in real life, conditions vary widely. For example, power-saving settings, poor network connections, or app interactions may interfere with the system’s performance. We did not study how users respond to failed attempts, repeated prompts, or account lockouts. Nor did we test procedures for helping users regain access or report errors during use.

The current study shows that the proposed authentication system is efficient and consistent across several mobile platforms. However, more testing is needed on a broader range of devices, under varied environmental and user conditions, and against more advanced security threats. Addressing these limitations will be key to improving the system’s reliability, fairness, and readiness for large-scale deployment in the future.

## 7. Conclusions

This study introduced a continuous authentication system designed for mobile devices. The approach combines facial recognition with environmental signals such as lighting and background noise. It also uses a lightweight, hash-based security protocol to ensure safe and fast verification. The system is built to work on devices with limited processing power and memory, and it protects user privacy by keeping biometric data on the device and sending only short, encrypted tokens during authentication.

One of the study’s central goals was to move beyond results tied to a single device. Many mobile security systems work well in specific test conditions but struggle in real-world settings with varied hardware. To address this, we tested the system on different smartphones that use different types of processors and operating systems. We kept the model and configuration the same for all devices and adjusted lighting and noise levels to simulate different environments. We used statistical methods to check if performance differences between devices were small enough to be considered practically equivalent. The results showed that the system’s speed, energy use, and accuracy remained consistent across devices, supporting its ability to generalize beyond the initial test environment.

The system’s simplicity is a strength. Instead of using complex cryptographic techniques that can be slow or energy-intensive, we used efficient hash functions and short verification tokens. The facial recognition model is small and optimized for mobile use. It processes a single frame quickly and uses very little power. Because the system adjusts how it uses signals based on the surrounding environment—giving less weight to unreliable inputs in poor conditions—it performs well even in low light or noisy settings. These design choices help ensure that the system works reliably across different types of mobile hardware.

In comparison to other security methods, this system finds a useful balance. Unlike some traditional encryption methods, it does not require long processing times. And unlike behavioral authentication systems, which often need extended observation periods (for example, to analyze walking patterns or typing speed), this system delivers rapid results with just one image and a few context checks. This makes it especially useful in situations where devices must conserve battery life or where quick decisions are important.

Because the system works consistently on different phones without needing device-specific adjustments, it can be deployed more easily and maintained at lower cost. It is suitable for environments where internet connections are limited or where strict data privacy rules apply. The design also supports consistent performance even under varied conditions, such as changes in lighting or background noise, which can affect other biometric systems.

However, while this study strengthens confidence in the system’s ability to generalize, some limitations remain. We tested only a small number of devices and users. Broader studies with more types of phones and more diverse user groups are needed. We did not test the system against all possible attack methods—for example, advanced spoofing techniques like realistic face masks or video replays. We also relied on tools built into operating systems to measure energy use, which are useful but not as precise as hardware-based tools.

Looking ahead, several directions stand out. Expanding the range of devices and user profiles will help ensure fairness and stability. Adding more signals—such as how a user interacts with their phone—may further improve performance in difficult conditions. Allowing devices to adapt locally over time without sharing user data, such as through federated learning, could improve accuracy while protecting privacy. These improvements will support broader deployment without losing the core strengths of speed, security, and efficiency.

## Figures and Tables

**Table 1:** Summary of the proposed face-recognition model architecture, showing the backbone structure (FaceNet-inspired ResNet-50), key layers, parameter counts, and compression strategies applied (depthwise separable convolutions, pruning, quantisation). The table highlights how the model achieves a compact design suitable for on-device continuous authentication while preserving recognition accuracy.

Component	Details
Input and preprocess	Face crop 112 × 112, RGB, mean-std normalization; detector confidence threshold d_t ≥ 0.85.
Backbone	4 conv layers + 2 dense; depthwise-separable; SE blocks.
Embedding	128-D, L2-normalized; cosine similarity for verification.
Params/MACs	3.9 M params; 0.43 G MACs @ 112 × 112.
Size	3.7 MB (FP32), 0.9 MB (INT8).
Compression	Quantization-aware training (QAT).
Latency/Energy	146 ms/6.8 mJ (FP32); 119 ms/5.2 mJ (INT8).
Metrics	LFW verification 96.3%; EER 3.7%; TPR@FAR = 10^−3^ = 91.2%.
Privacy	Embedding stays on-device; protocol uses 128-bit hashed token z_u (§see Section 3.8).

**Table 2:** Ablation results of the lightweight CNN model, comparing different compression strategies (baseline, depthwise separable convolutions, pruning, and quantisation) in terms of verification accuracy on the LFW dataset and median latency on the Snapdragon 778G testbed. Results highlight the trade-off between efficiency and recognition performance.

Variant	Params (M)	Size (MB)	MACs (G)	Latency (ms)	Energy (mJ)	LFW Verification (%)	EER (%)	TPR@FAR = 10^−3^
FP32	3.9	3.7	0.43	146	6.8	96.3	3.7	91.2
INT8 (QAT)	3.9	0.9	0.43	119	5.2	95.8	4.3	90.6

**Table 3:** Performance metrics of the proposed adaptive authentication protocol under three lighting levels (low ≤50 lux, medium 50–300 lux, bright >300 lux) and three ambient noise levels (quiet <35 dB SPL, moderate 35–45 dB SPL, noisy >45 dB SPL). Results are based on 2700 authentication cycles executed on the Snapdragon 778G test bed, reporting median latency, CPU utilization, memory footprint, and per-cycle energy consumption across environmental conditions.

Condition	Latency (ms)	CPU Utilization (%)	Energy per Cycle (mJ)	Notes
**Low Light (≤50 lux)**	44.7 ± 2.5	Perf: 29.6, Eff: 21.8	20.9	Slightly higher latency due to reduced facial image quality
**Medium Light (50–300 lux)**	43.1 ± 2.3	Perf: 28.2, Eff: 21.4	20.1	Stable performance: adaptive fusion maintains balance
**Bright Light (>300 lux)**	41.8 ± 2.1	Perf: 27.5, Eff: 20.9	19.7	Lowest latency: high-quality input reduces processing retries
**Quiet Noise (<35 dB SPL)**	42.5 ± 2.2	Perf: 28.1, Eff: 21.2	19.8	Baseline condition; most efficient operation
**Moderate Noise (35–45 dB SPL)**	43.6 ± 2.4	Perf: 28.6, Eff: 21.5	20.4	Minor increase due to re-checks triggered by audio fluctuations
**High Noise (>45 dB SPL)**	47.1 ± 2.6	Perf: 29.8, Eff: 22.0	21.3	More frequent re-authentication cycles; overhead still <10%

**Table 4:** Comparative performance metrics of the proposed adaptive protocol versus three baselines; Elliptic-Curve Diffie–Hellman (ECDH), Pre-Shared Key (PSK), and a non-adaptive variant of our system, evaluated on the Snapdragon 778G test bed. Metrics include median authentication latency, CPU utilization, memory footprint, and per-cycle energy consumption. Results average over 2700 authentication cycles under varying lighting and noise conditions. A ✓ indicates the protocol satisfies the corresponding security property, while ✗ indicates the property is not supported.

Metric	Proposed Protocol	ECDH Protocol	PSK Protocol
**Median Latency (ms)**	42.3 ± 2.1	61.2 ± 3.8	39.4 ± 1.9
**CPU Utilization (%)**	27.8 (perf.), 21.2 (eff.)	38.7 (perf.), 29.5 (eff.)	25.1 (perf.), 19.3 (eff.)
**Memory Footprint (MB)**	62.7 (peak) → 54.5 (steady)	71.2 (peak) → 65.4 (steady)	53.8 (peak) → 51.7 (steady)
**Energy per Cycle (mJ)**	19.8 (95th: 21.2)	26.4 (95th: 28.1)	18.5 (95th: 19.7)
**Handshake Data (bits)**	2560	3400+	2100
**Forward Secrecy**	✓	✓	✗
**Biometric Linkage**	✓	✗	✗

**Table 5:** Decision latency for HMOG/Touchalytics reflects the minimum window length required to accumulate evidence ~0 s). Message size accounts for feature vectors and tags in our measurement logging; it does not include TLS overhead (constant across methods).

Method	EER (%)	TPR@FAR = 10^−3^ (%)	Median Decision Latency (ms)	Energy/Decision (mJ)	Message Size/Decision (bits)
Proposed (Face + Env. Fusion)	3.7	91.2	42	19.8	2560
HMOG (IMU + Keystroke)	7.9	84.6	20,000	28.4	1200
Touchalytics-style Gestures	4.8	88.1	20,000	24.6	1000

**Table 6:** (**a**): The table lists device model, SoC, RAM, OS build, and inference delegate, together with median latency, energy per cycle, CPU utilization, and memory footprint with 95% bootstrap confidence intervals. Differences in hardware capabilities do not translate into material gaps in runtime or efficiency, supporting portability of the proposed pipeline across heterogeneous smartphones; (**b**): TOST equivalence for latency and energy relative to the Snapdragon 778G baseline. All devices meet the pre-specified margins with significant TOST results and negligible effect sizes, indicating operational parity across platforms. Confidence intervals were estimated with 10,000-sample BCa bootstrap.

(a)								
Device	SoC/Chipset	RAM (GB)	OS Build	Inference Delegate	Median Latency (ms, 95% CI, Bootstrap)	Median Energy (mJ, 95% CI, Bootstrap)	CPU Utilization (%)	Memory Footprint (MB)
Google Pixel 6 (Baseline)	Snapdragon 778G Kryo 670	8	Android 13 (Build TQ3A.230805.001)	TFLite + NNAPI (CPU/GPU/NPU)	42.3 (41.0–43.7)	19.8 (19.1–20.6)	17.2	48.5
Samsung Galaxy S22	Snapdragon 8 Gen 1	12	Android 13 (Build TP1A.220624.014)	TFLite + NNAPI (GPU/NPU)	41.7 (40.5–42.9)	19.5 (18.9–20.4)	16.8	49.0
Xiaomi Redmi Note 12 Pro	MediaTek Dimensity 1080	8	Android 12 (Build SKQ1.220303.001)	TFLite + NNAPI (CPU/GPU)	43.1 (42.0–44.6)	20.1 (19.4–21.0)	17.5	50.2
(**b**)								
Device	Metric	Δ vs. Baseline (Point Estimate)	95% CI (BCa bootstrap)	Equivalence Margin	TOST Statistic		*p*-value	Effect Size (Cliff’s δ)
Samsung Galaxy S22	Latency (ms)	–0.6	[–1.8, 0.7]	±10% (≈±4.2 ms)	–2.41		<0.01	–0.05
Samsung Galaxy S22	Energy (mJ)	–0.3	[–0.9, 0.4]	±1.5 mJ	–2.16		<0.05	–0.04
Xiaomi Redmi Note 12 Pro	Latency (ms)	+0.8	[–0.4, 2.1]	±10% (≈±4.2 ms)	–1.98		<0.05	0.06
Xiaomi Redmi Note 12 Pro	Energy (mJ)	+0.3	[–0.5, 1.0]	±1.5 mJ	–1.87		<0.05	0.05

Notes: Confidence intervals (95%) were obtained via 10,000-sample BCa bootstrap resampling. Device models and OS build numbers are included for reproducibility. All devices were tested with brightness fixed at 200 nits, airplane mode enabled, and device temperature <38 °C prior to runs. Measurements were based on ≥900 authentication cycles per device under controlled environmental conditions; Margins were pre-registered prior to experiments. Latency margin = ±10% of baseline median (42.3 ms); energy margin = ±1.5 mJ relative to 19.8 mJ baseline. Bootstrap confidence intervals derived from 10,000 replicates. Effect sizes close to zero indicate negligible practical difference.

**Table 7:** Comparative analysis of continuous-authentication modalities on mobile devices, including facial recognition, voice, gait, keystroke, and sensor-fusion approaches. Reported metrics cover authentication performance (accuracy/false reject rate), computational cost (latency, energy consumption), and contextual robustness (sensitivity to lighting, noise, and mobility). The table highlights trade-offs across modalities and positions of the proposed framework within the broader mobile authentication landscape.

Modality and Exemplar	Dataset/Scenario	Decision Window	Reported Performance	Latency/Energy/Data (Reported)	Notes
Touch/gesture (Touchalytics)—30 touch features from scroll gestures	Natural scrolling on smartphone; intra-/inter-session and 1-week tests	Not fixed; continuous strokes	Median EER: 0% (intra), 2–3% (inter), <4% at 1 week	Not reported	Strong in-session performance; best as part of multimodal continuous auth, not standalone long-term.
HMOG (IMU during typing)—hand micro-movements + taps/keystroke	100 users; sitting vs. walking; typing on virtual keyboard	Often ~20 s windows evaluated	EER: 7.16% (walking) and 10.05% (sitting) with HMOG + tap + keystroke fusion	Energy overhead: ≈7.9% at 16 Hz sampling (vs. 20.5% at 100 Hz)	Shows explicit accuracy–energy trade-off; performance improves during walking.
Inertial gait (IDNet)—pocket-phone acc/gyro, CNN + one-class SVM	Walking with phone in front pocket	<5 gait cycles fused	Misclassification <0.15% with fewer than five cycles	Not reported	Orientation-independent cycle extraction; deep features + multi-stage decision.
This work (vision + context fusion)	Mobile front-camera + light/audio/IMU/gaze/crowd; single-frame decisions	Frame-level (with EWMA smoothing)	Face verification (LFW): 96.3%; continuous-auth thresholding as in §3	Latency: ~42 ms per decision; Energy: ~19.8 mJ; Data: ~−24.7% vs. ECDH baseline	Low-latency/low-energy, privacy-preserving token binding; complementary to longer-window behavioral methods.

## Data Availability

The raw data supporting the conclusions of this article will be made available by the authors on request.
